# Kundalini Yoga Meditation Versus the Relaxation Response Meditation for Treating Adults With Obsessive-Compulsive Disorder: A Randomized Clinical Trial

**DOI:** 10.3389/fpsyt.2019.00793

**Published:** 2019-11-11

**Authors:** David Shannahoff-Khalsa, Rodrigo Yacubian Fernandes, Carlos A. de B. Pereira, John S. March, James F. Leckman, Shahrokh Golshan, Mário S.R. Vieira, Guilherme V. Polanczyk, Euripedes C. Miguel, Roseli G. Shavitt

**Affiliations:** ^1^BioCircuits Institute, University of California, San Diego, La Jolla, CA, United States; ^2^Center for Integrative Medicine, University of California, San Diego, La Jolla, CA, United States; ^3^The Khalsa Foundation for Medical Science, Del Mar, CA, United States; ^4^The National Institute of Developmental Psychiatry for Children and Adolescents (INPD), Department of Psychiatry, School of Medicine, University of São Paulo, São Paulo, Brazil; ^5^Mathematics and Statistics Institute, Statistics Department, University of São Paulo, São Paulo, Brazil; ^6^Department of Psychiatry and Behavioral Sciences, Duke School of Medicine, Durham, NC, United States; ^7^Child Study Center, Department of Pediatrics and Psychiatry, Yale University School of Medicine, New Haven, CT, United States; ^8^Department of Psychiatry, University of California, San Diego, La Jolla, CA, United States; ^9^Hospital Israelita Albert Einstein, São Paulo, Brazil

**Keywords:** Yale-Brown Obsessive Compulsive Scale, Dimensional Yale-Brown Obsessive-Compulsive Scale, mindfulness, mental health, anxiety/anxiety disorders, depression

## Abstract

**Background:** Obsessive-compulsive disorder (OCD) is often a life-long disorder with high psychosocial impairment. Serotonin reuptake inhibitors (SRIs) are the only FDA approved drugs, and approximately 50% of patients are non-responders when using a criterion of 25% to 35% improvement with the Yale-Brown Obsessive-Compulsive Scale (Y-BOCS). About 30% are non-responders to combined first-line therapies (SRIs and exposure and response prevention). Previous research (one open, one randomized clinical trial) has demonstrated that Kundalini Yoga (KY) meditation can lead to an improvement in symptoms of obsessive-compulsive severity. We expand here with a larger trial.

**Design:** This trial compared two parallel run groups [KY vs. Relaxation Response meditation (RR)]. Patients were randomly allocated based on gender and Y-BOCS scores. They were told two different (unnamed) types of meditation would be compared, and informed if one showed greater benefits, the groups would merge for 12 months using the more effective intervention. Raters were blind in Phase One (0–4.5 months) to patient assignments, but not in Phase Two.

**Main Outcome Measures:** Primary outcome variable, clinician-administered Y-BOCS. Secondary scales: Dimensional Yale-Brown Obsessive Compulsive Scale (clinician-administered), Profile of Mood Scales, Beck Anxiety Inventory, Beck Depression Inventory, Clinical Global Impression, Short Form 36 Health Survey.

**Results:** Phase One: Baseline Y-BOCS scores: KY mean = 26.46 (SD 5.124; N = 24), RR mean = 26.79 (SD = 4.578; N = 24). An intent-to-treat analysis with the last observation carried forward for dropouts showed statistically greater improvement with KY compared to RR on the Y-BOCS, and statistically greater improvement on five of six secondary measures. For completers, the Y-BOCS showed 40.4% improvement for KY (N = 16), 17.9% for RR (N = 11); 31.3% in KY were judged to be in remission compared to 9.1% in RR. KY completers showed greater improvement on five of six secondary measures. At the end of Phase Two (12 months), patients, drawn from the initial groups, who elected to receive KY continued to show improvement in their Y-BOCS scores.

**Conclusion:** KY shows promise as an add-on option for OCD patients unresponsive to first line therapies. Future studies will establish KY’s relative efficacy compared to Exposure and Response Prevention and/or medications, and the most effective treatment schedule.

**Clinical Trial Registration:** www.ClinicalTrials.gov, identifier NCT01833442.

## Introduction

Obsessive-Compulsive Disorder (OCD) is defined by intrusive, unpleasant, and recurrent thoughts (obsessions) that are often recognized to be irrational and excessive, and are often accompanied by repetitive behaviors (compulsions) performed in an attempt to eliminate the anxiety caused by the obsessions (1). A recent US survey found that about 25% of adults reported experiencing obsessions or compulsions at some point in their lives, and 2.3% met DSM-IV lifetime prevalence criteria, with a 12-month occurrence of 1.2% (2). Severe impairments with a high psychosocial impact, pronounced suffering, decreased quality of life, and substantial financial costs are associated with the morbidity and treatment of OCD (3, 4). The World Health Organization reports that OCD is one of the ten most disabling disorders worldwide (5).

Both behavioral and pharmaceutical interventions have demonstrated efficacy. Exposure and response prevention (ERP) is considered to be the most efficacious cognitive behavioral therapy (CBT) for treating OCD (6). The efficacy of ERP for OCD has been evaluated in various meta-analyses in recent decades, reporting large effects sizes in comparison to waiting list groups (mean d = 1.30) (7). When comparing against active control conditions, like relaxation training, ERP continued to show large effect sizes (mean d = 1.18) (8). A 2015 review of ERP and cognitive therapy (CT) added that the methods of delivery are important, with *in vivo* therapist assisted ERP, in conjunction with imagery, producing the greatest change in symptom severity (6).

Serotonin reuptake inhibitors (SRIs) and clomipramine are considered to be the effective pharmacological treatments (9, 10). However, owing to better tolerability, selective serotonin reuptake inhibitors (SSRIs) are the pharmacological treatment of choice (10). Soomro et al., conducted a meta-analysis of SSRIs versus placebo, and found in 17 studies (3,097 patients) that SSRIs are nearly twice as likely as placebo to produce a response when using a more stringent criteria of >25% reduction in the Yale-Brown Obsessive Compulsive Scale (Y-BOCS) (11). Discontinuation is often associated with a relapse and a decrease in the patient’s quality of life, thus necessitating of long-term treatment that often includes significant side effects. About a third of patients fail SSRIs (9).

Here we address how Kundalini Yoga (KY) meditation techniques have been tested previously in an 11-part “OCD-specific” protocol that includes eight primary techniques and three optional techniques (12, 13). Two studies have examined the efficacy of this protocol in treating OCD: one open uncontrolled trial (12) and a randomized controlled trial (RCT) comparing the KY meditation protocol against Relaxation Response (RR) plus the Mindfulness Meditation (13). The 12-month open pilot trial (12) started with eight patients (seven females), with a Y-BOCS baseline mean of 21.125 (SD +4.32). Five patients were medicated, and their medications were stable for >3 months prior to entry. All of these patients had attempted medication and ERP prior to enrollment. Five completed the trial and improved on the Y-BOCS with a group mean improvement of 54%, (Y-BOCS mean = 8.80; SD +6.98). Twelve months later, four of the five medicated patients were off medication for periods ranging from 9 to 19 months with lasting improvements. The RCT (13) compared parallel run groups of the same KY protocol (11 adults, 1 adolescent, mean baseline Y-BOCS score 22.75; SD + 5.15) against the RR + Mindfulness Meditation techniques (10 adults, mean baseline Y-BOCS score 22.80; SD +5.39). Patients were informed that two protocols with multiple meditations would be compared without naming the types of meditation or describing the techniques. Patients remained blind to the names and contents of the other protocol during the controlled phase. Seven adults in each group completed 3 months of therapy and the KY group demonstrated a significantly greater improvement on the Y-BOCS (*P* < 0.047; mean group differences were KY = 9.43, SD + 7.21; RR = 2.86, SD + 3.13). An intent-to-treat (ITT) analysis with the last observation carried forward (LOCF) with the Y-BOCS for the baseline and 3-month tests showed that only the KY group improved. At 15 months, the final merged KY group (n = 11) improved 71% (mean endpoint Y-BOCS = 6.6; SD +6.33).

The aim of this RCT is to conduct an efficacy trial and replication with this KY meditation protocol in a more rigorous and larger sample of adults with OCD. Our hypothesis is that treatment with KY would show significantly more reduction in the overall severity of OCD compared to the RR on the Y-BOCS and other measures [Dimensional Yale-Brown Obsessive Compulsive Scale (DY-BOCS, clinician-administered), Profile of Mood Scales (POMS), Beck Anxiety Inventory (BAI), Beck Depression Inventory (BDI), Clinical Global Impression (CGI, clinician administered), Short Form 36 Health Survey (SF-36)].

## Methods

### Setting

Treatment (March 2012 to July 2013) was conducted at the Obsessive-Compulsive Spectrum Disorders Program, Department of Psychiatry, University of São Paulo School of Medicine, Brazil. Patients were recruited by advertisements. The institutional review board at the University of São Paulo approved the study in compliance with the Code of Ethics of the World Medical Association (Declaration of Helsinki). All patients signed a consent form after the study was explained.

### Participants

**Figure 1:**
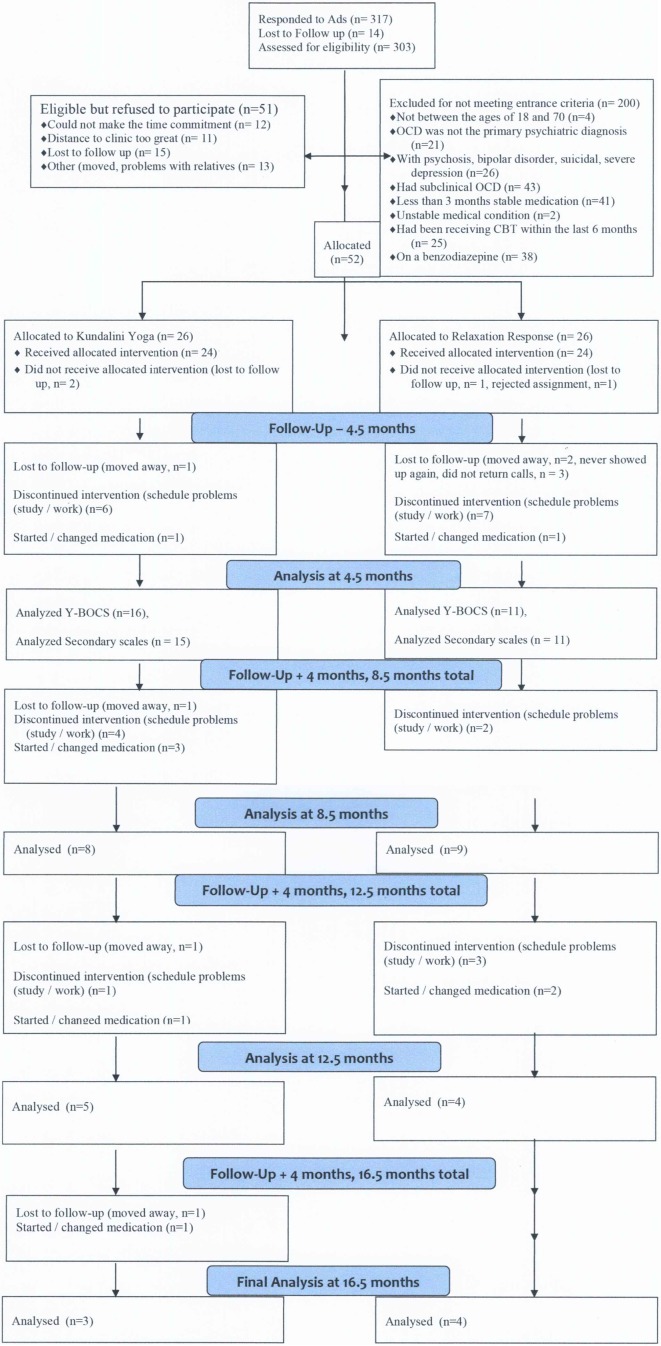
Consolidated standards of reporting trials diagram. Flow of patients through the study. OCD, obsessive compulsive disorder; CBT indicates cognitive-behavioral therapy.

Fifty-two patients were randomized and 24 entered treatment in each group. Patients, ages 18 to 65 years (mean 41.67, SD +12.89), were screened for eligibility by trained raters with expertise in OCD and related disorders, including DSM-IV-R diagnosis of OCD that had to be present for >6 months, and >16 on the Y-BOCS, (see **Figure 1**; CONSORT diagram). Patients on medication had to be stabilized for 3 months prior to entry and informed that they could not change medication(s) during the trial or increase their dose, although they could reduce their dose. No compensation beyond free treatment was provided. They were informed the trial might last 12 to 24 months. Patients were blind to the protocol names of both groups and to their content prior to the start of therapy. They were also blind to the name and content of the other group during Phase One (0–4.5 months). They were informed if one intervention showed greater efficacy, groups would merge for 12 months using the more beneficial intervention. Raters (AMR, SB) were blind to patient assignments in Phase One; Phase Two (4.5 to 16.5 months) was open.

Patients were excluded for smoking, substance abuse problems, psychoactive prescription medications other than those for OCD, spinal problems, or other physically limiting problems (excessively overweight, seizure disorders, pulmonary or cardiovascular disorders); major depressive disorder (MDD) with psychotic features, schizophrenia, bipolar disorder, mental retardation, anorexia nervosa, bulimia nervosa, autism spectrum disorders, traumatic brain injury, without regular and reliable transportation, choose not to participate in meditation and/or chanting (out loud or silently) for personal or religious reasons, or if they were undergoing or had undertaken psychotherapy, ERP, or CT for OCD in the previous six months.

### Randomization

Patients were randomly allocated (1:1) into two groups (KY or RR) using a computer-generated stratified block allocation procedure (14) that balanced the treatment groups for gender and Y-BOCS scores. The name and content of the group intervention for the respective group was only revealed at the start of treatment. KY was run on Thursday at 5:00 to 7:00 PM, and RR on Tuesday at 3:00 to 5:00 PM, based on room and therapist availability. Subjects were told which day and time they would attend by the study coordinator prior to treatment. Those that could not attend their group allocation due to work and scheduling conflicts were allowed to switch to the other group. Nine allocated to each group requested a switch, prior to knowing the name and content of either group.

### Treatments

Protocols were practiced in chairs. The KY group was led by a physician certified as a KY teacher (RFY). A clinical psychologist and RR practitioner led the RR group (Marcelo Camargo Batistuzzo, PhD). Neither had previous experience running groups in a clinical trial. The 11-part KY protocol is described in complete detail (15–19). One of the primary techniques is claimed to be specific for treating OCD. All were taught on day 1, and all but an elective technique (12) for anger were practiced routinely in the group. Skills and the length for practice developed over time. Patients were requested to practice at home every non-group day and had the option to practice 1 to 10 or all 11. Total practice time is 65 min for techniques 1 to 10. The revised version of the RR meditation (20) was taught and practiced for three 20-min rounds in the RR group. They were instructed to practice for 60 min on non-group days. Patients in both groups were given sheets to mark their times spent each day on each of their respective techniques. Weekly attendance was monitored and recorded by group instructors. Both protocols are included in complete detail in the **Supplement**.

### Main Outcome Measures and Assessments

Evaluations for OCD, other psychiatric disorders, inclusion/exclusion criteria, medication status, prior CBT/ERP/CT, as well as the three clinician-administered assessments: Y-BOCS, DY-BOCS, CGI, were conducted by trained clinicians (AMR, SB) specialized in treating OCD patients.

The Y-BOCS (21, 22) was employed to assess OCD severity. The Y-BOCS rates symptoms over the previous week and includes ten items: five assessing obsessions and five assessing compulsions severity (time, interference, distress, resistance, control). Each item is scored using a Likert scale ranging from 0 to 4, with a total score ranging from 0 to 40.

The DY-BOCS (23) rates OCD symptoms over the previous week on 88 items with six dimensions: harm-aggression, sexual-religious, contamination-cleaning, symmetry-ordering-repeating-counting, hoarding, and miscellaneous (includes somatoform and OCD-spectrum disorders symptoms). The clinical severity of each dimension is evaluated and includes an overall severity score (time, interference, distress), with a maximum total score of 30.

The CGI (24) was used to assess the severity of OCD symptoms. Its scores range from 1 (minimal) to 7 (very severe).

The POMS (25) assesses distinct mood states over the past week and includes 65 items rated using a five-point Likert scale ranging from 0 (not at all) to 4 (extremely), for: Tension-Anxiety, Depression-Dejection, Anger-Hostility, Vigor-Activity, Fatigue-Inertia, and Confusion-Bewilderment. A sum of the six items represents the Total Mood Disorder (TMD) value.

The BAI (26) and the BDI (27, 28) are each 21-item self-report questionnaires that assess symptom severity for anxiety and depression, respectively. Both scales have a total score range from 0 to 63.

The SF-36 (version 2.0) (29) includes an 8-scale profile of functional health and well-being scores as well as psychometrically-based physical and mental health summary measures and a preference-based health index.

The clinician-administered Systemic Assessment for Treatment Emergent Events was used to monitor for events. This measure includes 26 items, rated as absent, mild, moderate, or severe (30).

### Statistical Methods

Descriptive statistics and exploratory graphing were used to assess the normality of the data for skew and/or outliers. Data was also examined for missing values and dropout rates. Baseline differences between groups, dropouts, and completers were also examined using Univariate Analysis of Variance (ANOVA) for the Y-BOCS, DY-BOCS, POMS, BAI, BDI, and SF-36. In Phase One, data analyses were performed following the ITT method with the LOCF for dropouts for all measures, excluding the CGI scale and SF-36. The Y-BOCS total score was the primary outcome measure. A completer’s analysis was conducted for all measures for Phase One and Two. The main end-point used to compare the two interventions for the completers analysis was the percent Improvement, defined by Im = (X-Y)/X, with X as the baseline and Y the measure at the end of each evaluation period. Continuous variables were analyzed using the standard methods of t-student, ANOVA, and regression methods. For categorical data, the χ2 test was used. With the SF-36 and POMS instruments, higher scores indicate greater improvement. For these two scales the positions of X and Y were reversed in the numerator to measure the improvement to help maintain consistency in the direction where a higher percentage reflects greater improvement.

For Phase Two, the merged group was analyzed using Bayesian inference. The parameter of interest here is the proportion of positive responses for each instrument. The posterior density was calculated for these parameters. The objective is to calculate the probability of the population proportion that is higher than a possible proportion value *P* (see formula in Methods in **Supplementary Material**).

All statistical tests were 2-tailed. Differences were considered statistically significant provided a *P* value of 0.05 or less is obtained. Statistical software included IBM SPSS Statistics (version 24) and Microsoft Excel for Mac 2011 (version 14.7.4).

## Results

### Sample

The CONSORT Diagram is shown in **Figure 1**. The demographics and clinical characteristics of the sample are presented in **Table 1**. There were no significant group differences at baseline regarding demographic and clinical characteristics, including the frequencies of the different OCD symptom dimensions in both groups, as assessed by the DY-BOCS symptom checklist. These results support our claim that the experiment is well balanced in respect to the patient demographics. The dropout rate in Phase One for those that received the allocation was 33% for KY and 54% for RR. The difference was not significant (χ2 = 0.8325, *P* = 0.362).

**Table 1:** Demographics and Clinical Characteristics of the Sample.

Characteristic	Kundalini Yoga(n = 24)	Relaxation Response(n = 24)	All(n = 48)
Female, No. (%)	18 (75.55)	13(54.17)	31 (64.58)
Age, y, mean (SD)	43.29 (13.97)	40.04 (11.80)	41.67 (12.89)
Age at OCD onset, y, mean (SD)	10.0 (5.28)	11.21 (8.93)	10.60 (7.39)
Education completed^a^ mean (SD)	2.542 (0.58)	2.625 (0.66)	2.583 (0.61)
Non-Hispanic white, No. (%)	21 (87.50)	19 (79.12)	40 (83.33)
Marital status, No. (%)			
Single	11 (45.83)	13 (54.17)	24 (50.00)
Married/partnered	10 (41.67)	9 (37.50)	19 (39.58)
Divorced/separated	3 (12.50)	2 (8.33)	5 (10.42)
Employed, No. (%)	14 (58.33)	19 (76.17)	33 (68.75)
Family income (Reals/month), mean (SD)	6,854.17 (5886)	5,733.33 (3681)	6,293.75 (4889)
Families with children, No., (%)	9 (37.5)	4 (16.67)	13 (27.08)
Religion, No., (%)			
Catholic	12 [31]	15 (62.5)	27 (56.25)
Spiritualist	3 (12.5)	6 (25)	9 (18.75)
Evangelical	0	2 (8.33)	2 (8.33)
Other	9 (37.5)	1 (4.2)	10 (20.83)
Duration of OCD, y, mean (SD)	33.29 (12.98)	28.83 (15.44)	31.06 (14.30)
Baseline Y-BOCS obsession score, mean (SD)	12.58 (2.48)	13.25 (2.40)	12.92 (2.44)
Baseline Y-BOCS compulsion score, mean (SD)	13.87 (3.22)	13.54 (3.02)	13.71 (3.09)
Baseline Y-BOCS total score, mean (SD)	26.46 (5.12)	26.79 (4.58)	26.62 (4.81)
Baseline DY-BOCS total score, mean (SD)	19.71 (3.33)	19.71 (3.83)	19.71 (3.55)
DYBOCS-1 Aggression, No. (%)	21 (87.50)	18 (75.00)	39 (81.25)
DYBOCS-2 Sexual/Religious, No. (%)	17 (70.83)	19 (79.17)	36 (75.00)
DYBOCS-3 Symmetry/Order, No. (%)	22 (91.67)	22(91.67)	44 (91.67)
DYBOCS-4 contamination/Cleaning, n (%)	19 (79.17)	18 (75.00)	37 (77.08)
DYBOCS-5 hoarding, n (%)	17 (70.83)	16 (66.67)	33 (68.75)
DYBOCS-6 miscellaneous^b^, n (%)	22 (91.67)	21 (87.50)	43 (89.58)
Current axis 1 diagnoses, n (%)			
OCD only	8 (33.33)	10 (41.67)	18 (37.50)
Depressive disorder (actual)	9 (37.50)	7 (29.17)	16 (33.33)
Other anxiety disorder (actual)	9 (37.50)	11 (45.83)	20 (41.67)
Depressive disorder (past)	2 (8.33)	4 (16.67)	6 (12.50)
Other anxiety disorder (past)	3 (12.50)	3 (12.50)	6 (12.50)
Medication, n (%)			
None	13 (54.17)	10 (41.67)	23 (47.92)
Current SSRI, n (%) mg/d per patient			
Citalopram	2 (8.33), 60, 40	1 (4.17), 60	3 (6.25)
Escitalopram	1 (4.17), 40	1 (4.17), 10	2 (4.17
Fluoxetine	1 (4.17), 40	1 (4.17), 80	2 (4.17)
Fluvoxamine	0	3 (12.5), 225, 100, 300	3 (6.25)
Paroxetine	1 (4.17), 20	2 (8.33), 20, 40	3 (6.25)
Sertraline	2 (8.33), 150, 150	1 (4.17), 150	3 (6.25)
Current SNRI, n (%) mg/d per patient			
Desvenlafaxine	0	1 (4.17), 50	1 (2.08)
Duloxetine	0	1 (4.17), 60	1 (2.08)
Venlafaxine	1 (4.17), 75	0	1 (2.08)
Current TCA, n (%) mg/d per patient			
Agomelatine	0	1 (4.17), 25	1 (2.08)
Amitriptyline	0	1 (4.17), 25	1 (2.08)
Clomipramine	4 (16.67), 100, 75, 50, 300	0	4 (8.33)
Nortriptyline	0	1 (4.17), 125	1 (2.08)
Others, No. (%) mg/d per patient			
Bupropion	0	1 (4.17), 150	1 (2.08)
Carbamazepine	0	1 (4.17), 200	1 (4.17)
Promethazine	0	1 (4.17), 25	1 (4.17)
Risperidone	1 (4.17), 1	0	1 (2.08)
Topiramate	1 (4.17), 150	0	1 (2.08)
Number of patients on two medications	3 (12.5)	3 (12.5)	6 (12.5)
Weight (kg), mean (SD)	63.28 (14.33)	75.05 (18.96)	69.11 (17.66)
Height (m), mean (SD)	1.65 (0.095)	1.70 (0.091)	1.67 (0.096)

No standard deviation (SD) is provided in those cells where n = 1; y = year, SSRI, selective serotonin reuptake inhibitor; SNRI, Serotonin–norepinephrine reuptake inhibitors; TCAs, Serotonin–norepinephrine reuptake inhibitors; Reals, Brazilian dollars

^a^A score of 1 would indicate the patient did not complete high school, 2 would indicate not completing college, and 3 indicate completing college.

^b^Includes somatoform and OCD spectrum disorders symptoms

Patients that attended at least one session of the allocated intervention are included in an analysis to first test for non-partiality or bias for the Y-BOCS and DY-BOCS. The Y-BOCS baseline means were compared for the following four groups at the end of Phase One: (1) 16 KY participants; (2) 8 KY dropouts; (3) 11 RR participants; (4) 13 RR dropouts. Their respective mean Y-BOCS baseline scores were 26.25 (SD +4.74); 26.88 (SD +6.15); 27.55 (SD +4.20); 26.15 (SD +4.95). There were no significant two-way interaction or main effects for group or dropout factors (Two-way: *f*_1.44_ = 0.47, *P* = 0.497). One KY patient moved immediately after taking the Y-BOCS at 4.5 months before taking the secondary scales.

The DY-BOCS means for the respective four groups were n = 15, 19.93 (SD +3.88); n = 9, 19.33 (SD +2.29); n = 11, 20.09 (SD +3.36); n = 13, 19.38 (SD +4.29). Similarly, there were no significant two-way interaction or main effects for group or dropout factors (Two-way: *f*_1.44_ = 0.01, *P* = 0.99). There were no significant baseline group differences for completers and dropouts for the POMS, BAI, and BDI.

### Efficacy of Treatment With an ITT LOCF Group Analysis—Phase One

#### Analysis for the Y-BOCS and DY-BOCS—Phase One (0 Month Baseline to 4.5 Months)

**Figure 2:**
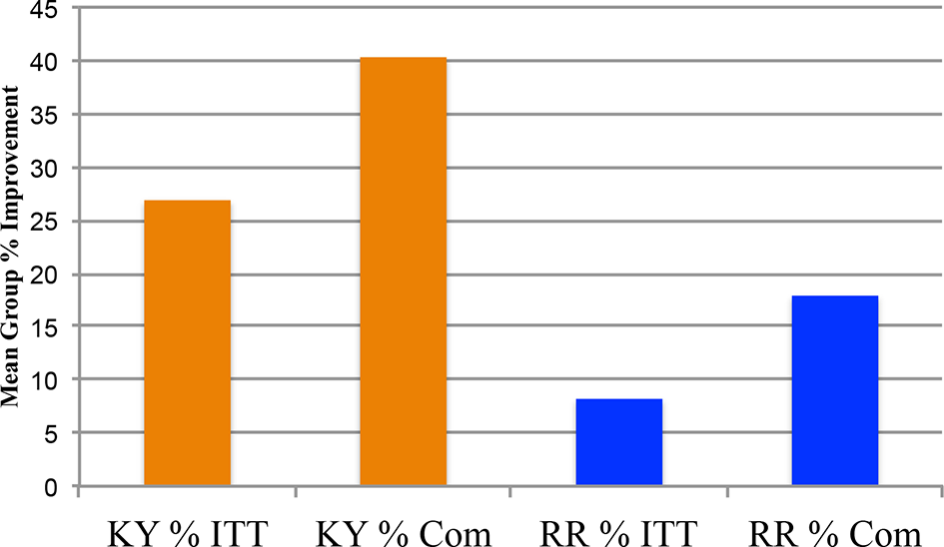
Y-BOCS total scores Phase One: 0 month and 4.5 months % changes: intent-to-treat and completers. The Phase One clinician-administered Yale-Brown Obsessive Compulsive Scale (Y-BOCS) Total Scores Group % changes: 0 month vs. 4.5 months, are plotted for the intent-to-treat (ITT) analysis using the last observation carried forward (LOCF), and for the completers (Com) for the Kundalini Yoga meditation group and Relaxation Response control group. The Kundalini Yoga ITT mean group % improvement was 26.90% (SD = ± 27.628%; *n* = 24). The Relaxation Response ITT mean group % improvement was 8.214% (SD = ± 13.137; *n* = 24). The Univariate Analysis of Variance for the ITT LOCF indicates that Kundalini Yoga had a greater % improvement on the Y-BOCS, (*f*_1.46_ = 8.96, *P* = 0.004). The Com Kundalini Yoga baseline mean was 26.25 (SD ±4.74; *n* = 16) and the 4.5-month mean was 15.19, showing a 40.4% (SD ±24.32%) mean group improvement. The Relaxation Response baseline was 27.55 (SD ±4.204; *n* = 11) and 22.45 (SD ±4.34; *n* = 11) at 4.5 months. The Com Relaxation Response group mean % improvement was 17.92% (SD = ± 14.34%; *n* = 11). The univariate analysis of variance for the completers indicates that Kundalini Yoga had a greater improvement on the Y-BOCS (*f*_1.25_ = 7.50, *P* = 0.011).

**Figure 3:**
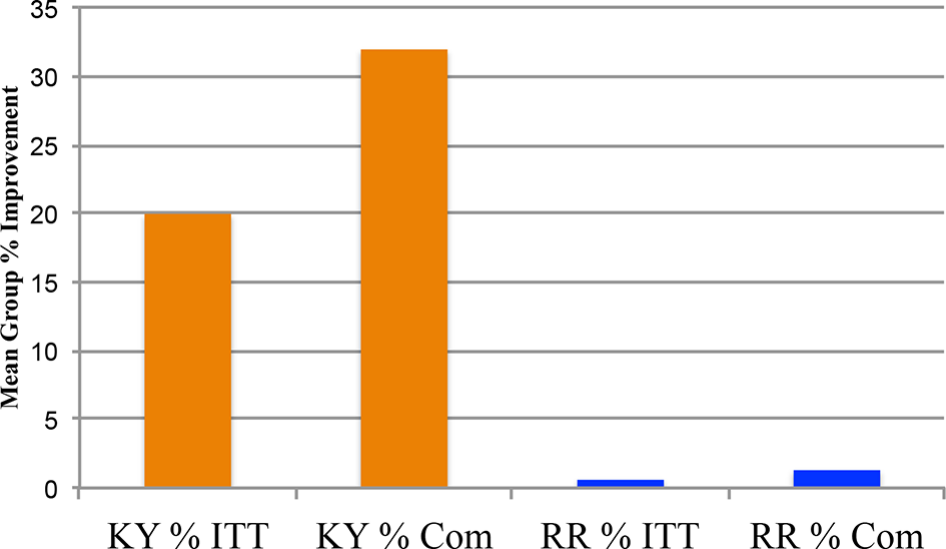
DY-BOCS total scores Phase One: 0 Month and 4.5 Months % Changes: Intent-To-Treat and Completers. The Phase One clinician-administered Dimensional Yale-Brown Obsessive Compulsive Scale (DY-BOCS) Total Scores Group % changes: 0 month vs. 4.5 months, are plotted for the intent-to-treat (ITT) analysis with the last observation carried forward (LOCF), and for the completers (Com) for the Kundalini Yoga meditation group and the control group Relaxation Response meditation technique. The Kundalini Yoga ITT mean group % change improvement was 19.98% (SD = ± 27.629%; *n* = 24). The Relaxation Response ITT mean group % change improvement was 0.592% (SD = ± 12.282%; *n* = 24). The Univariate Analysis of Variance for the ITT LOCF indicates that Kundalini Yoga had a greater mean group % change improvement on the DY-BOCS, (*f*_1.46_ = 9.86, *P* = 0.003)]. The Com baseline means for Kundalini Yoga and Relaxation Response are 19.93 (SD ±4.46; *n* = 15) and 20.09 (SD ±3.36; *n* = 11), respectively. The 4.5-month means for Kundalini Yoga and Relaxation Response are 13.33 (SD ±5.81; *n* = 15) and for 19.55 (SD ±3.75; *n* = 11), respectively. The Com Kundalini Yoga group mean % improvement was 31.969% (SD ±29.04%; *n* = 15). The Com Relaxation Response group mean % improvement was 1.298% (SD ±18.599%; *n* = 11). The Univariate Analysis of Variance for the Com indicates that Kundalini Yoga had a greater improvement on the DY-BOCS, (*f*_1.24_ = 9.384, *P* = 0.005).

Only baseline and 4.5-month measures were taken. The KY group baseline mean was 26.46 (SD +5.124; *n* = 24) and the RR baseline mean was 26.79 (SD +4.578; *n* = 24). There was a significant (*f*_1.46_ = 8.96, n = 24, *P* = 0.004, Eta Square = 0.163, Power = 0.834) difference between the two groups (KY n = 24, RR n = 24) for the Y-BOCS mean % change improvement (KY: 26.902%, SD +27.63%; RR: 8.214%, SD +13.14%), see **Figure 2**. For the DY-BOCS the KY group baseline mean was 19.71 (SD +3.329; n = 24) and the RR baseline mean was 19.71 (SD +3.828; n = 24). Similarly, there was a significant (*f*_1.46_ = 9.86, n = 24, *P* = 0.003, Eta Square = 0.177, Power = 0.868) difference between the two groups for the DY-BOCS mean % change improvement (KY: 19.98%, SD +27.63%; Relaxation Response: 0.595%, SD +12.28%), see **Figure 3**.

#### Analysis for the POMS, BAI, and BDI—Phase One (0 Month Baseline to 4.5 Months)

**Figure 4:**
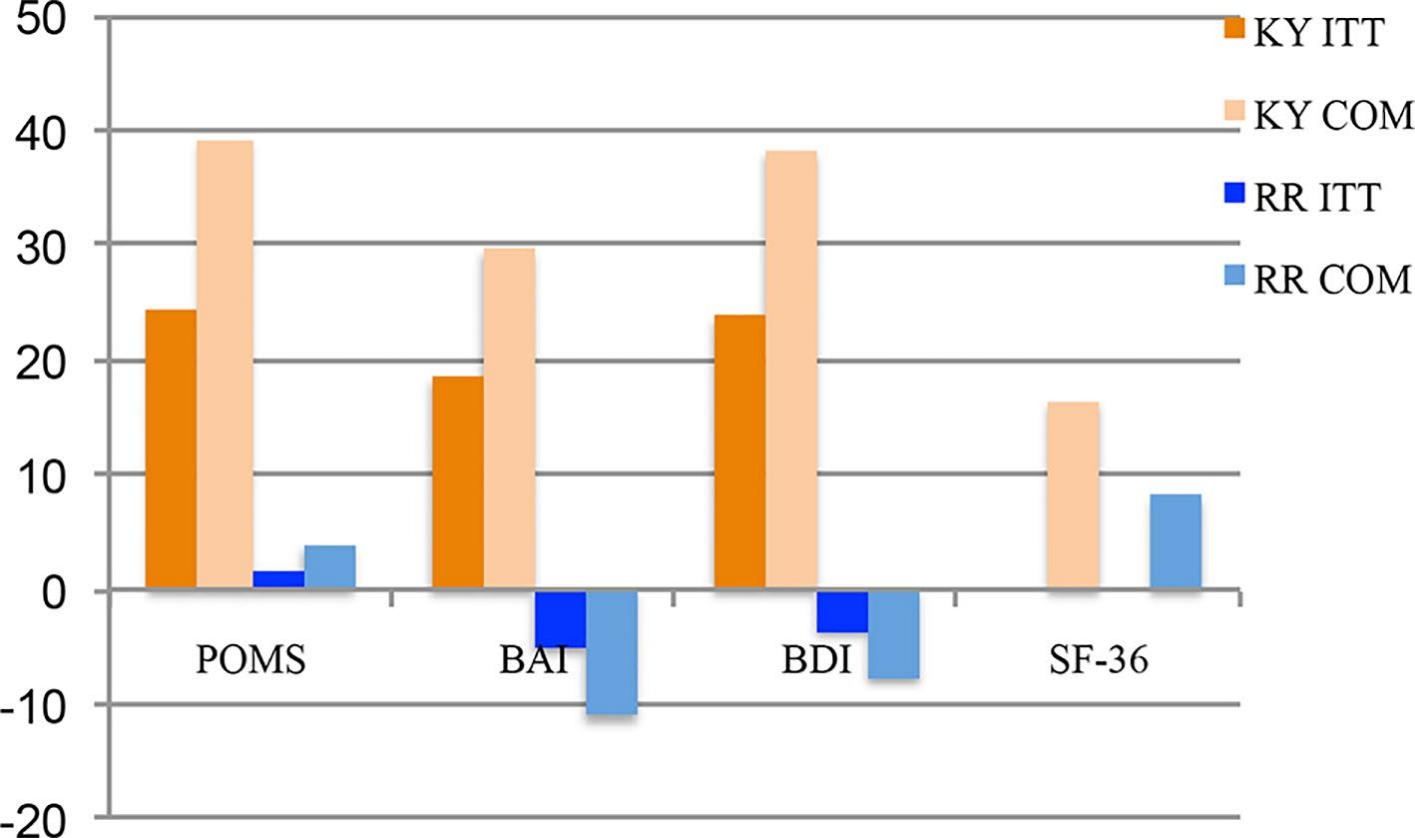
POMS total mood disorder scores, BAI scores, BDI scores, and SF-36 scores for Phase One: 0 month and 4.5 months % changes: intent-to-treat and completers. **Figure 4**shows the Profile of Moods States Total Mood Disorder (POMS TMD), the Beck Anxiety Index (BAI), the Beck Depression Inventory (BDI) scores, and the Short Form Health Survey (SF-36) scores for the Phase One % change improvement scores for the Kundalini Yoga and Relaxation Response groups when comparing the 0-month and 4.5-month scores. The POMS intent-to-treat (ITT) analysis with the last observation carried forward (LOCF) Kundalini Yoga group (0-month mean = 122.083; 4.5-month mean = 101.958) mean change score was −20.125 (SD ± 31.208; n = 24) and −0.709 (SD ± 15.398; n = 24) for the Relaxation Response group (0-month mean, 113.5; 4.5-month mean 112.791; n = 24). This difference of 19.417 was significant (P = 0.01). The ITT LOCF POMS mean % change improvement for 0 month to 4.5 months was 24.452% (SD ±37.72%; n = 24) for the Kundalini Yoga group and 1.773% (SD ± 15.38%, n = 24) for the Relaxation Response group. This difference was significant P = 0.009. The completer (Com) group baseline mean for Kundalini Yoga is 127.533 (SD ± 50.141; n 15) and 116 (SD ± 45.29; n = 11) for the Relaxation Response. The Com group 4.5-month mean for Kundalini Yoga is 95.33 (SD ± 36.976; n = 15) and 114.454 (SD ± 47.922; n = 11) for the Relaxation Response. The mean group % change score for Kundalini Yoga improvement was 39.123% (SD ± 41.505%; n = 15) and 3.869% (SD ± 23.138%; n = 11) for the Relaxation Response. The group differences are significant, (f1.25 = 6.42, P = 0.018). (**Figure 4**) Also shows the BAI % change improvement scores for the Kundalini Yoga and Relaxation Response groups. The ITT analysis with the LOCF for the BAI mean change score for 0 to 4.5 months was −4.00 (SD ± 5.741; n = 24) for the Kundalini Yoga group [0-month mean 17.708 (SD ± 8.094; n = 24); 4.5-month mean 13.708; (SD ± 8.379; n = 24)] and −0.708 (SD ± 4.982; n = 24) for the Relaxation Response group [0-month mean 15.375; (SD ± 11.336; n = 24); 4.5-month mean 14.667 (SD ± 10.98; n = 24)]. This change score difference of 3.292 was significant (2-tail) P = 0.039. The ITT LOCF BAI mean % change improvement for 0 to 4.5 months was 18.52% (SD ± 32.23%; n = 24) for the Kundalini Yoga group and –5.10% (SD ±42.86%; n = 24) for the Relaxation Response group. This difference was significant (2-tail) P = 0.036. The Com group baseline mean for Kundalini Yoga is 17.33 (SD ±7.724; n = 15) and 16.0 (SD 11.983; n = 11) for Relaxation Response. The completer group 4.5-month mean for Kundalini Yoga is 10.93 (SD ±6.766; n = 15) and 14.45 (SD ±11.228; n = 11) for Relaxation Response. The mean % change score improvement was 29.63% (SD ± 36.799%; n = 15) and –11.13% (SD ± 64.429%; n = 11) for Relaxation Response. The group differences are significant, (f_1.24_ = 4.2, P = 0.05, 2-tail). (**Figure 4**) Also shows the BDI % change scores for the Kundalini Yoga and Relaxation Response groups for the baseline to 4.5 months. The ITT analysis with the LOCF for the BDI mean change score for 0 to 4.5 months was −7.042 (SD ± 10.564; n = 24) for the Kundalini Yoga group [0-month mean 21.0 (SD ± 10.505); 4.5-month mean 13.958 (SD ± 8.518)] and –0.333 (SD ± 3.293; n = 24) for the Relaxation Response group [0-month mean, 17.208 (SD ±10823); 4.5-month mean, 16.875 (SD ± 10.617)]. This change score difference of 6.708 was significant P = 0.005. The BDI mean % change improvement for 0 to 4.5 months was 23.90% (SD ± 41.27%; n = 24) for the Kundalini Yoga group and −3.53% (SD ± 25.54%; n = 24) for the Relaxation Response group. This difference was significant (P = 0.008). The Com group baseline mean for Kundalini Yoga is 22.67 (SD ±11.81; n = 15) and 17.18 (SD ±10.84; n = 11) for the Relaxation Response. The Com group 4.5-month mean for Kundalini Yoga is 11.4 (SD ± 8.16; n = 15) and 16.45 (SD ± 10.35; n = 11) for the Relaxation Response. The group differences are significant, (f1.24 = 7.05, P = 0.014). The BDI Com Kundalini Yoga mean % change improvement was 38.242% (SD ± 47.012%; n = 15), and –7.705% (SD ± 38.278%; n = 11) for the Relaxation Response. Group differences are significant (f1.24 = 7.05, P = 0.014). **Figure 4** also shows the SF-36 Com Scores Phase One 0 to 4.5 months % Changes. The Com group baseline mean for Kundalini Yoga is 89.73 (SD ± 22.53, n = 15) and 83.73 (SD ± 16.29, n = 11) for the Relaxation Response. The Com 4.5-month mean for Kundalini Yoga is 73 (SD ± 17.96, n = 15) and 76.91 (SD ± 20.11, n = 11) for the Relaxation Response. The Com mean Kundalini Yoga % change improvement was 16.4% (SD ± 3.87%; n = 15) and 8.1% (SD ± 5.98%; n = 11) for the Relaxation Response. The group differences are not significant (P = 0.18)

For the POMS, the KY group baseline mean was 101.96 (SD +40.058; n = 24) and the RR baseline mean was 112.79 (SD +45.223; n = 24). The KY (n = 24) and RR (n = 24) groups were significantly different (*f*_1.46_ = 7.44, *P* = 0.009, Eta Square = 0.139, Power = 0.761) for the POMS mean % change improvement (KY: 24.452%, SD +37.72%; RR: 1.773%, SD +15.38%), see **Figure 4**. For the BAI, the KY group baseline mean was 17.71 (SD +8.093; n = 24) and the RR baseline mean was 15.38 (SD +11.336; n = 24). The groups were significantly different (*f*_1.46_ = 4.66, *P* = 0.036, Eta Square = 0.092, Power = 0.561) for the BAI mean % change improvement (KY: 18.52%, SD +32.23%; RR: −5.10%, SD +42.86%), see **Figure 4**. For the BDI, the KY group baseline mean was 21.00 (SD +10.505; n = 24) and the RR baseline mean was 17.21 (SD +10.823; n = 24). The group difference was also significant (*f*_1.46_ = 7.67, *P* = 0.008, Eta Square = 0.143, Power = 0.744) for the BDI (KY: 23.90%, SD +41.27%; RR: −3.53%, SD +25.54%), see **Figure 4**. An ITT LOCF analysis was not conducted on the SF-36 since the completer’s analysis showed no statistical group difference.

### Efficacy of Treatment With the Completer’s Group Analysis—Phase One

#### Y-BOCS Scores—Phase One (0 Month Baseline to 4.5 Months)

**Figure 2** shows the Y-BOCS total mean % changes at 4.5 months for the KY and RR Response completer groups. The KY group 0-month baseline mean was 26.25 (SD +4.74; n = 16) and 15.19 (SD +5.86; n = 16) at 4.5 months, with a mean % change improvement of 40.4% (SD +24.32%; n = 16). The RR 0-month baseline mean was 27.55 (SD +4.20; n = 11) and 22.45 (SD +4.34; n = 11) at 4.5 months, with a mean % change improvement of 17.9% (SD +14.34%). The two groups were significantly different on their mean % change improvement (*f*_1.25_ = 7.50, *P* = 0.011, Eta Squared = 0.231, Power = 0.749).

#### Secondary Outcome Scales—Phase One (0 Month Baseline to 4.5 Months)

Phase One outcomes for the secondary scales are reported for the DY-BOCS, POMS, BAI, BDI, SF-36, and CGI. Valid data were available for 15 KY and 11 RR patients for the six secondary scales. One KY patient moved immediately after taking the Y-BOCS at 4.5 months before taking the secondary scales.

**Figure 3** shows the DY-BOCS mean % change improvement for the KY and RR groups at 4.5 months. The 0-month baseline means for KY and RR are 19.93 (SD +4.46; n = 15) and 20.09 (SD +3.36; n = 11), respectively. The 4.5-month means for KY and RR are 13.33 (SD +5.81; n = 15) and for 19.55 (SD +3.75; n = 11), respectively. The mean % change KY improvement was 31.969% (SD +29.04%), and 1.298% (SD +18.6%) for the RR. Group differences are significant (*f*_1.24_ = 9.384, *P* = 0.005, Eta Squared = 0.281, Power = 0.836).

**Figure 4** shows the POMS TMD mean % change improvement scores. The baseline group mean for KY was 127.533 (SD +50.141; n = 15) and 116 (SD +45.29; n = 11) for the RR. The 4.5-month mean for KY is 95.33 (SD +36.976; n = 15) and 114.454 (SD +47.922; n = 11) for RR. The KY mean % change improvement was 39.123% (SD +41.505%; n = 15) and 3.869% (SD +23.138%; n = 11) for RR. Group differences are significant (*f*_1.25_ = 6.42, *P* = 0.018, Eta Squared = 0.211, Power = 0.682).

**Figure 4** also shows the BAI mean % change improvement scores. The baseline group mean for KY is 17.33 (SD +7.724; n = 15) and 16.0 (SD +11.983: n = 11) for the RR. The completer 4.5-month mean for KY is 10.93 (SD +6.766; n = 15) and 14.45 (SD +11.228; n = 11) for RR. The BAI KY mean % change improvement was 29.634% (SD +36.799%; n = 15) and −11.13% (SD +64.429%; n = 11) for RR. Group differences are significant (*f*_1.24_ = 4.2, *P* = 0.05, Eta Squared = 0.149, Power = 0.502).

**Figure 4** also shows the BDI mean % change improvement scores. The baseline group mean for KY is 22.67 (SD +11.81; n = 15) and 17.18 (SD +10.84; n = 11) for RR. The completer 4.5-month mean for KY is 11.4 (SD +8.16; n = 15) and 16.45 (SD +10.35; n = 11) for RR. The BDI KY mean % change improvement was 38.242% (SD +47.012%; n = 15), and −7.705% (SD +38.278%; n = 11) for RR. Group differences are significant (*f*_1.24_ = 7.05, *P* = 0.014, Eta Squared = 0.227, Power = 0.722).

**Figure 5:**
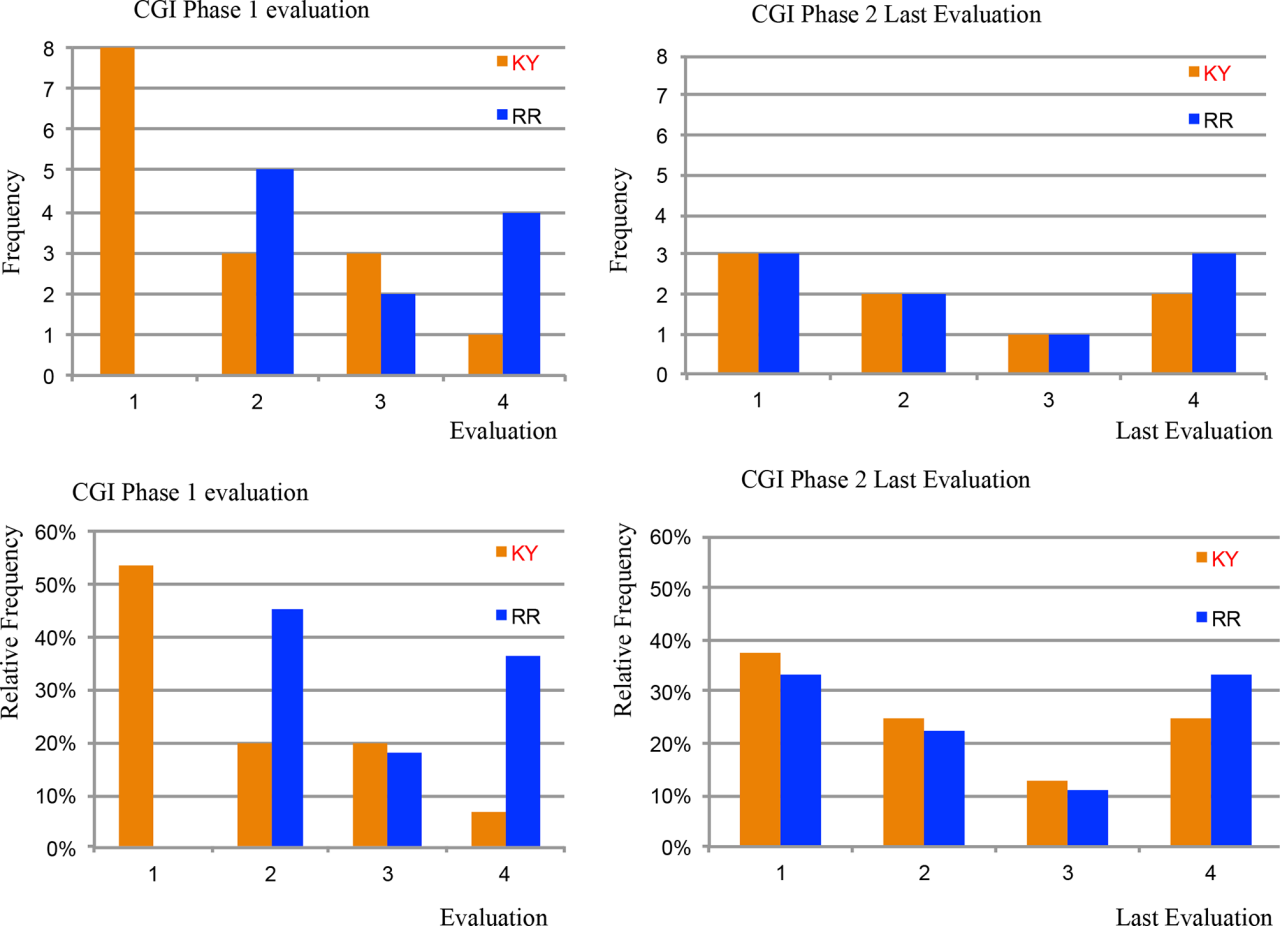
CGI Scores in Phases One and Two. **Figure 5** shows the clinician-administered Clinical Global Impression Scale (GCI) scores for both Kundalini Yoga and the Relaxation Response for Phase One and Two for both the frequency and the relative frequency in %’s for the four possible scores of 1, 2, 3, or 4. At 4.5 months Kundalini Yoga had eight scores of 1, and 7 with a 2, 3, or 4, and the Relaxation Response had 0 scores of 1, and 11 with a 2, 3, or 4. Kundalini Yoga shows greater clinical improvement compared to the Relaxation Response with the distributions of 1’s versus 2, 3, or 4 (*P* = 0.007). The Kundalini Yoga group had only one 4, and 14 values of a 1, 2, or 3, and the Relaxation Response had four 4’s, and seven values of a 1, 2, or 3. This difference was also significant with *P* = 0.034. Phase Two did not show statistical differences when comparing the patients that were originally in the Kundalini Yoga group vs those originally in the Relaxation Response group (See **Table S1** in Supplement).

**Figure 5** shows the CGI scores for both KY and RR for Phase One and Phase Two for both the frequency and the relative frequency in %’s for the four possible scores of 1, 2, 3, or 4. **Table S1** (in **supplements**) shows the results for the Statistical Homogeny Exact Test for the CGI scores when comparing the frequency of values of scoring 1 versus 2, 3, or 4 and the frequencies of scoring 4 versus 1, 2, or 3 for KY and RR. At 4.5 months KY has eight scores of 1, and 7 with a 2, 3, or 4, and RR had 0 scores of 1, and 11 with a 2, 3, or 4. For Phase One KY shows greater clinical improvement compared to RR with the distributions of 1’s versus 2, 3, or 4, *P* = 0.007. The KY group had only one 4, and 14 values of a 1, 2, or 3, and RR had four 4’s, and 7 values of a 1, 2, or 3. This difference was also significant (*P* = 0.034).

### Efficacy of Treatment for Phase Two—Y-BOCS and Secondary Scales

#### Y-BOCS Scores—Phase Two (4.5 to 16.5 Months)

**Figure 6:**
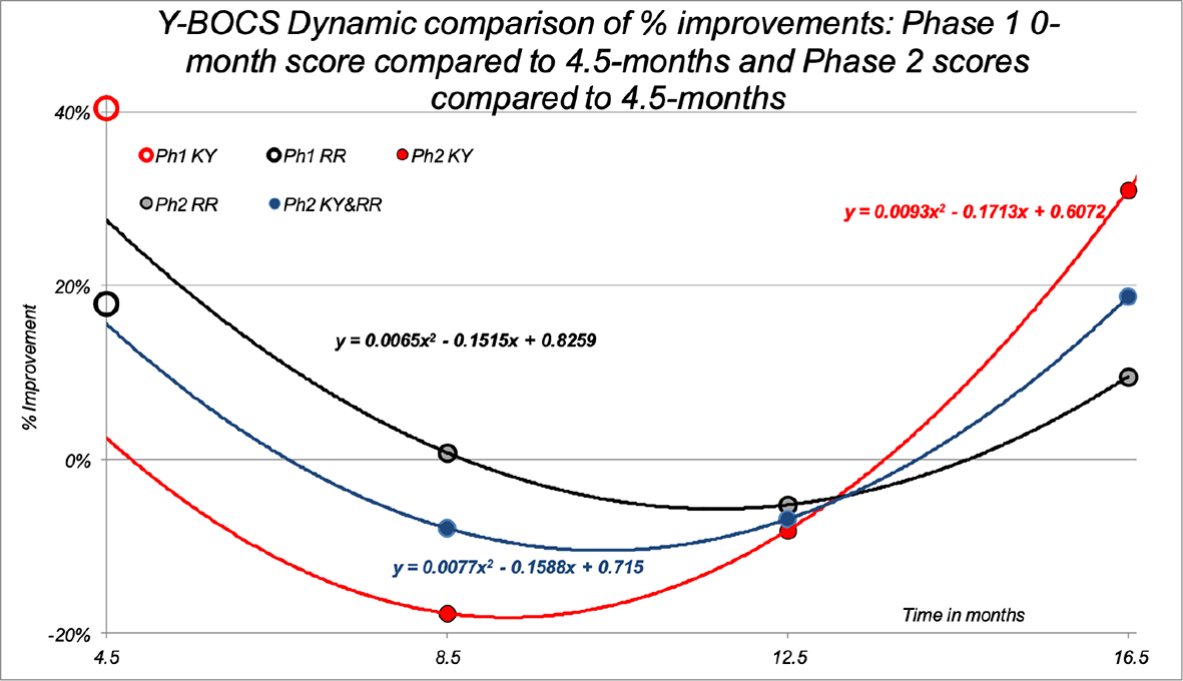
Phase Two Y-BOCS Total % improvement. **Figure 6** shows the Y-BOCS mean % change improvement for the patients remaining at 8.5 months (n = 17), 12.5 months (n = 9), and 16.5 months (n = 7) for those who were in their original groups (red = KY, black = RR), and also for the patients combined in the KY Phase Two group (blue = combined). Of the seven that completed the 16.5-month time point, three were from the original KY group and 4 from the RR group. The additional % mean change improvement for the seven subjects when comparing their 4.5-month and 16.5-month means was 18.76% (SD +36.91%), with a 31.07% (SD +36.65%) improvement for the three originally from KY and 9.53% (SD +7.53%) for the 4 originally from RR. When comparing the three patients originally from KY at 16.5 months to their Phase One 0-month baseline score, they had a 50.62% (SD +42.35%) mean % change improvement. The four RR patients had a 26.96% (SD +21.94%) overall improvement, and all seven combined showed an overall improvement of 37.1% (SD +37.91%). For reference, **Figure 6** also shows the mean % improvement plotted for the Y-BOCS Total Phase One 4.5-month % improvement scores plotted on the y-axis for the completers for the KY meditation group (n = 16) with an open red circle and the RR control group (n = 11) with an open black circle. The KY mean showed a 40.4% improvement, and the RR mean was 17.9%.

**Figure 6** shows the Y-BOCS mean % change additional improvement for the patients remaining at 8.5 months (n = 17), 12.5 months (n = 9), and 16.5 months (n = 7) for those in their original groups, and for the combined group. For the seven that completed the 16.5-month time point, 3 were from the original KY group and four were from the RR group. The % mean change improvement for the seven subjects when comparing their 4.5-month and 16.5-month means was 18.76% (SD +36.91%), with a 31.07% (SD +36.65%) improvement for the three originally from KY and 9.53% (SD +7.53%) for the four originally from RR. When comparing the three patients originally from KY at 16.5 months to their Phase One 0-month baseline score, they had a 50.62% (SD + 42.35%) mean % change improvement. The four RR patients had a 26.96% (SD +21.94%) overall improvement, and all seven combined showed an overall improvement of 37.1% (SD +37.91%).

**Figure 7:**
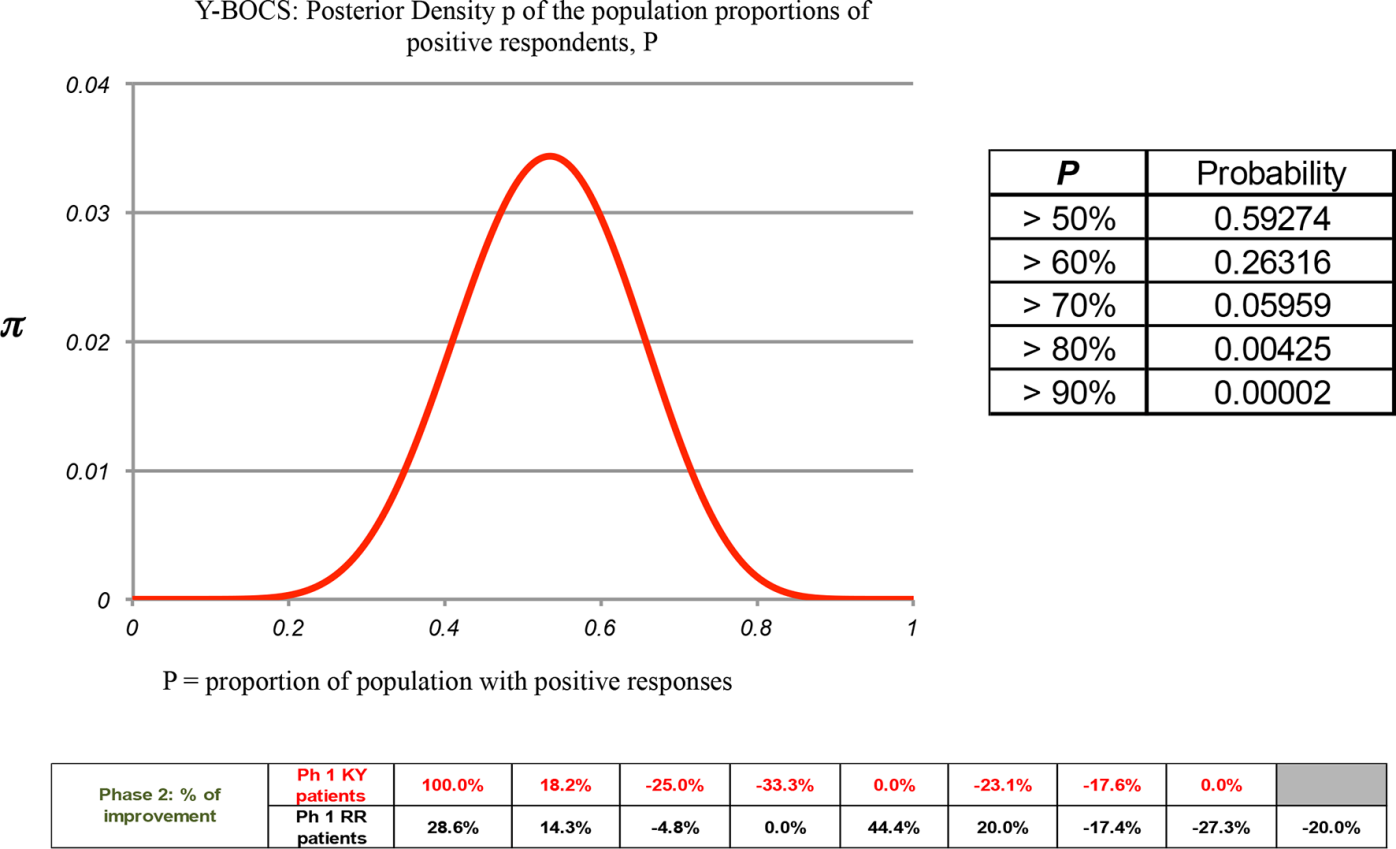
A Phase Two Bayesian analysis for the Y-BOCS: The calculation of the Posterior Density π of the population showing the proportion of positive respondents, P. A Bayesian statistical analysis was performed for 17 patients comparing their 4.5-month value with their last Y-BOCS measure taken at dropout that included the seven that completed the 16.5-month trial end point. This analysis showed that a 50% or greater probability criteria of patient improvement in the Y-BOCS in Phase Two was *P* = 0.593, where 0 = no patients improving and 1 = all patients improving.

Due to the significant dropout of patients for the long Phase Two 12-month period, a Bayesian statistical analysis was performed for the 17 patients comparing their 4.5-month value with their last Y-BOCS measure that included the seven that completed the 16.5-month trial end point, see **Figure 7**. This analysis showed that using a criteria of a 50% or greater probability of any patient improving on the Y-BOCS in Phase Two was *P* = 0.593, where 0 = no patients improving and 1 = all patients improving.

#### Secondary Outcome Scales—Phase Two (4.5 Months to 16.5 Months)

Phase Two outcomes for the DY-BOCS, POMS, BAI, BDI, and SF-36, are shown in **Figures S1–S5** (in **Supplement**), respectively. These five Figures show the 8.5-month (n = 17), 12.5-month (n = 9), and 16.5-month (n = 7) means when compared to their 4.5-month mean scores for those from their original Phase One groups separately and all patients combined in the Phase Two KY group.

**Figure S1** shows the DY-BOCS mean % change improvement. The mean % change improvement for the seven subjects when comparing their 4.5- and 16.5-month means was 26.22% (SD + 37.23%, n = 7), with a 38.96% (SD +52.69%) improvement for the three from KY and 16.67% (SD +11.43%) for the four from RR. The DY-BOCS mean % improvement for the seven completers at 16.5 months compared to the Phase One 0-month baseline was 32.8% (SD +36.11%). The three original KY improved by 51.02% (SD +42.5%), and the four original RR patients by 20.79%, SD +8.98%).

**Figure S2** shows the POMS mean % change improvement. The mean % improvement for the seven completers was 21.13% (SD ±8.33%), with a 24.24% (SD ±14.87%) improvement for the three from KY, and 18.81% (SD ±23.44%) for the four from RR. The POMS % improvement for the seven completers at 16.5 months compared to the Phase One baseline was 32.92% (SD ±16.52%). The three original KY improved by 41.05% (SD ±16.13%), and the 4 original RR patients by 26.83% (SD ±14.0%).

**Figure S3** shows the BAI mean % change improvement. The mean % improvement for the seven completers was 8.03% (SD +53.46%), with a 24.12% (SD +54.43%) improvement for the 3 from KY, and −4.05% (SD +49.38%) for the 4 from RR. The BAI % improvement for the seven completers at 16.5 months compared to the Phase One baseline was 2.11% (SD +86.42%). The three original KY patients improved by 34.29% (SD +38.14%), and the 4 original RR patients regressed by −22.03% (SD +103.06%).

**Figure S4** shows the BDI mean % change improvement. The mean % improvement for the seven completers was 45.28% (SD + 36.49%). The three original KY patients improved by 36.19% (SD + 42.68%), and the 4 original RR patients by 52.09% (SD + 19.93%). The BDI % improvement for the seven completers at 16.5 months compared to the Phase One baseline was 58.64% (SD +21.99%). The three original KY improved by 71.96% (SD + 22.69%), and the 4 original RR by 48.65% (SD +15.08%).

**Figure S5** shows the SF-36 mean % change improvement. The mean % improvement for the seven completers was 2.05% (SD + 15.57%). The three original KY patients improved by 15.51% (SD +7.69%), and the original 4 RR patients by −8.05% (SD + 11.93%). The SF-36% improvement for the seven completers at the 16.5 months compared to the Phase One baseline was 15.05% (SD + 11.98%). The three original KY improved by 19.22% (SD + 8.69%), and the 4 original RR by 11.92% (SD + 13.1%).

The Phase Two results for the CGI scale are shown in **Figure S5**and **Table S1** (in **Supplement**) for the 17 patients that completed at least the first CGI scale measure, i.e., the 8.5-month mark. In Phase Two the original KY subjects improved more than the original RR subjects across all six secondary measures.

A Bayesian statistical analysis for Phase Two was employed for the DY-BOCS, POMS, BAI, BDI, and SF-36 for the patients as a single group (KY + RR) comparing their 4.5-month value with their last measure taken at drop out, including those that completed the trial at 16.5 months (see **Figures S6–S10**, respectively; in **Supplement**). The probability P of >50% of the patients improving to any extent is P = 0.994, 0.881, 0.952, 0.76, 0.407, respectively for the five instruments, where 0 = no patients improving and 1 = all patients improving.

### Protocol and Treatment Adherence for Completers

#### Attendance

The Phase One KY weekly group mean attendance rate for completers (n = 16) was 63.38% (range 50%–84%) and for the RR completers (n = 11) the attendance rate was 71.45% (range, 50%–100%). For Phase Two, the completer rate was 69.76% for months 4.5 to 8.5, (range, 54%–100%, n = 17); 71.33% for months 8.5 to 12.5 (range, 50%–100%, n = 9), and 75% for months 12.5 to 16.5 (range, 50%–90%, n = 7).

#### Homework

Basing the weekly homework rate on a maximum practice time of 75 min/day for KY and 60 min/day for RR, the Phase One mean percent rate of homework completion for KY was 23.27% (range 0% to 82.31%, n = 16), and for RR the rate was 18.67% (range 0% to 65.27%, n = 11). For Phase Two, months 4.5 to 8.5, the percent was 35.56% (range 0% to 130.90%, n = 17); for months 8.5 to 12.5, the percent was 55.65% (range 0% to 154.11%, n = 9); and for months 12.5 to 16.5, the percent was 51.65% (range 0% to 127%, n = 7).

### Adverse Effects

No treatment or other adverse events were reported in Phase One or Phase Two on the clinician-administered Systemic Assessment for Treatment Emergent Events.

## Discussion

This randomized trial shows superior efficacy for KY as compared to RR in the treatment of OCD in adults. An ITT LOCF analysis showed the superiority of KY on the primary outcome measure (Y-BOCS) and the DY-BOCS, POMS, BAI, and BDI. Subjects continued to improve in the Phase Two open label extension, however, with a significant dropout rate. Both treatments were well tolerated. The findings in this trial are comparable to the prior open trial (12) and the randomized trial (13) using this KY protocol for treating OCD.

For subjects that completed, the extent of improvement for KY relative to RR was highly clinically significant. All of the final KY completer groups had a reduction of >35% on the Y-BOCS, with the exception of the four subjects in the subgroup that started with RR that had only a 27% improvement at the end of Phase Two. The 35% level is an accepted threshold for a positive treatment response when the response is defined as much or very much improved on the CGI-I (28, 29). When symptom remission is defined as having mild to no symptoms on the CGI and a Y-BOCS score of <12 (29) our results show significant merit and are close to the two earlier KY trials that had final Y-BOCS mean scores of 8.8 (12) and 6.6 (13). If we use the criteria of a Y-BOCS of <12, for the trial reported here, there were six patients with end points <12. In addition, there were three 0 scores on the DY-BOCS. For the five of eight participants who completed the open trial, there were 4 patients with scores <12 (12). The previous RCT, with 11 completers, had 8 scores <12.

A completer analysis also showed KY superior compared to RR for all of the secondary measures (DY-BOCS, POMS, BAI, BDI, and CGI) in Phase One, with the exception of significance for SF-36. However, the findings on the secondary measures are to be considered provisional until they are replicated with a larger sample. In the first RCT (13) the completer KY group also improved significantly more than the RR + Mindfulness Meditation control on the Y-BOCS with a 38.38% improvement compared to 13.9% for RR + Mindfulness Meditation for the 3-month controlled phase, and also with significantly greater improvement on the secondary measures with the POMS, Symptoms Checklist List-90-Revised-Obsessive-Compulsive Scale (SCL-90-R-OC), Symptoms Checklist List-90-Revised-Global-Severity-Index Scale (SCL-90-R-GSI), and with non-significant but greater improvements on the Perceived Stress Scale (PSS) and Purpose-in-Life (PIL) test. The first RCT Phase One KY 3-month improvements for the secondary measures were 47.68% (SCL-90-R-OC), 49.44% (SCL-90-R-GSI), 62.41% (POMS), 30.05% (PSS), and 10.60% (PIL) (13). The respective % improvements for RR + Mindfulness Meditation were –3.87%, 0.63%, –2.51%, 8.92%, and –1.10%.

We have observed a similar distribution of OCD symptoms based on the DY-BOCS subgroups here at baseline, which allows us to consider that the different outcomes cannot be attributed to variance in OCD symptom profiles between groups. For example, hoarding symptoms have been associated with a poorer response in pharmacological and cognitive-behavioral treatments (31). Here, the frequency of hoarding symptoms was similar in both groups.

In Phase Two we had a large and unexpected patient dropout rate over the various time points, which complicated the more standard approaches to statistical analysis. Only 27% of the patients that finished Phase One completed Phase Two. Therefore, a Bayesian analysis was performed to calculate the significance for any improvements for a proportion of the population. The probability *P* of >50% of the population improving in Phase Two was *P* = 0.593 (Y-BOCS), 0.994 (DY-BOCS), 0.881 (POMS), 0.952 (BAI), 0.76 (BDI), 0.407 (SF-36), where 0 = no patients improving and 1 = all patients improving.

The content in these two meditation protocols seems to account for the differences in the benefits observed here and in the other two trials. KY includes one technique that yogis had discovered in ancient times that was claimed to be effective for treating OCD (32, 33). This OCD-specific technique is in the class of yogic breathing practices called unilateral forced nostril breathing (UFNB), and is a highly structured four-phase left nostril specific pattern (see technique eight in the protocol, in **Supplement**). The differential physiological and psychological effects of the less complex left and right UFNB techniques have been reviewed (34, 35). However, the OCD-specific UFNB pattern has demonstrated broad global affects across the right cerebral hemisphere when studied with dual 37-channel magnetoencephalography (35). We postulate that these broad right hemispheric effects may account for the therapeutic value of this technique. The other components in this OCD-specific KY protocol are included to help increase the patients´ ability to more quickly gain temporary relief and to accomplish mastery of the “OCD-specific” technique. The other KY techniques also help to manage symptoms that often accompany obsessions and compulsions and this may help explain why the POMS, BAI, and BDI also showed group differences.

To the best of our knowledge only two other studies have been published comparing a yoga protocol (36) or one that included meditation (37) for the treatment of OCD. The 10-day 2-week uncontrolled Hatha yoga pilot states “we could not find any literature on validating a specific yoga module for OCD.” Hence, they created a 22-part protocol that includes two chanting techniques, five standard Hatha yoga breathing techniques, 14 hatha yoga exercises, and a final 7-min rest period. The total practice time for their module was 1 h. They recruited 17 patients, 13 completed the first week, and 10 completed the second week. Of the 10 to complete, they eliminated one as an outlier due to a high Y-BOCS baseline score of 35 and a high 2-weeks score of 28. With the remaining nine, they report significant improvement on the clinician-administered Y-BOCS and CGI scale. Also, Mindfulness-based cognitive therapy was compared to a wait-listed control group in an 8-week trial that was employed after the completion of a CBT intervention for OCD patients who continued to suffer from significant symptoms (37). They used the Y-BOCS Self‐Report as their primary efficacy variable. The intervention group (N = 18) went from 24.18 to 21.69 (change score, –2.49) and the wait-listed group (N = 18) went from 25.35 to 26.76 (change score +1.41).

Future trials can investigate whether a one or two week lead in intensive can help increase the retention rate and the overall benefits of therapy. Various schedules may also help determine the best course of therapy. However, the KY protocol requires months to perfect.

### Limitations

One limitation of this trial is the final Phase Two sample size. This is due in part to the 16.5-month study time, where attrition can occur for many reasons. The socio-economic stressors: long working days, insufficient public transportation, and heavily-congested São Paulo traffic may have also led to dropouts. The fairly demanding homework assignments may also have contributed to the dropouts. Another potential factor contributing to dropout was that patients were blinded to the types of meditation prior to entry, and few had any prior meditation experience. Also, there was no randomization for Phase Two. In contrast, our strengths were that we were able to run two parallel-matched groups (N = 24 per group), with strict inclusion/exclusion criteria. While the metropolitan region of the city of São Paulo had a population near 21 million, it took us a full year to recruit patients that matched our criteria that remained eligible at the start. We were also able to show a significant difference in the efficacy of the two treatments on all scales with the exception of the SF-36, using both a strict ITT LOCF and completer’s analysis. However, since nine patients in each group, prior to knowing the content or name of either protocol, switched groups for time and day convenience prior to the start of therapy, this may have added an unknown factor to group differences. Nonetheless, at baseline the groups were nearly identical for their Y-BOCS and DY-BOCS scores. In addition, no Y-BOCS or DY-BOCS differences in baseline scores were noted between the patients that continued to the end of Phase One or the dropouts for Phase One. The secondary measures and other demographic measures also did not have significant baseline differences.

### Generalizability

The patients recruited for this trial were “real-world” patients commonly seen in any academic out-patient treatment center, with comorbid anxiety and MDD, un-medicated or medicated on a SSRI, SNRI, TCA, or other medications known to be helpful in treating OCD. However, it may be argued that the strict inclusion/exclusion criteria, that also eliminated smokers, may reduce the generalizability of our patient population. Their mean age of OCD onset was 10.6 years, and the illness duration was 31.06 years prior to treatment. Many of the patients had also previously attempted medication and/or CBT/ERP without adequate success. Our sample has been treated in a tertiary hospital, consisting of patients with early onset OCD and a long duration of illness. For this reason, our results may not be easily translatable to a community sample with milder symptoms.

### Clinical Implications

The benefits of KY meditation for adults with OCD have now been demonstrated in three clinical trials (one uncontrolled trial, two RCTs). Some clinicians may wish to consider KY as an alternative option if CBT/ERP and/or medications are not a desired patient choice, or if they are already medicated and/or taking ERP and their therapies fail to provide satisfactory relief. If medications and CBT/ERP have failed to provide an adequate resolution of obsessions and compulsions, then KY might well be attempted prior to any invasive therapy, including gamma knife surgery and deep brain stimulation, which are currently considered as last resorts. Also, some clinicians may consider adding this KY protocol to all patients with the disorder along with first line approaches in order to amplify treatment response.

## Data Availability Statement

Clinical Trial Registration: www.ClinicalTrials.gov, identifier NCT01833442.

## Ethics Statement

The institutional review board at the University of São Paulo approved the study in compliance with the Code of Ethics of the World Medical Association (Declaration of Helsinki). All patients signed a consent form after the study was explained.

## Author Contributions

Access to raw data: RF, CP (the senior statistician) had full access to all of the data in the study and take responsibility for the integrity of the data and the accuracy of the data analysis. Study concept and design: DS-K, JM, JL, CP, EM, and RS. Acquisition of data: RF and RS. Analysis and interpretation of data: All authors. Drafting of the manuscript: All authors. Critical revision of the manuscript for important intellectual content: All authors. Statistical analysis: CP, SG, DS-K, RS, and RF. Obtained funding: EM, GP, RS, JL, JM, and DS-K.

## Funding

This study was supported by the National Institute of Developmental Psychiatry for Children and Adolescents (INPD), CNPq–grant no. 573974/2008-0; and State Government–Sao Paulo State Foundation for Research Support (FAPESP); grant no. 2008/57896-8. The sponsors of this trial had no input or influence on the study design, the collection, analysis and interpretation of data, or in the writing of the report, and in the decision to submit the article for publication.

## Conflict of Interest

DS-K reports royalties from two books published by W.W. Norton & Co, Inc. that includes the Kundalini Yoga meditation protocol, and personal sales for a DVD for the protocol, and OCD patient fees. The protocol is also published in complete detail in the **Supplement** and free online (see 16). He has no other conflicts of interest and has not received grant support for this trial. JM is a professor of psychiatry and behavioral sciences, Emeritus, at the Duke University School of Medicine, Durham, NC. He has no conflicts of interests. SG certifies that he has no affiliations with or involvement in any organization or entity with any financial interest or non-financial interest in the subject matter or materials discussed in this manuscript. EM, RS, GP, CP, and RF are affiliated with the institution (University of Sa~o Paulo) that received grant support for the work, and certify that they have no other conflicts of interests. GP also reports personal fees from Shire, Teva, and Medice, outside the submitted work. JL and MV certify they have no conflicts of interest.

## References

[1] Abramowitz JS Taylor S McKay D Obsessive-compulsive disorder. Lancet (2009) 374:491–9. 10.1016/S0140-6736(09)60240-3 19665647

[2] Ruscio AM Stein DJ Chiu WT Kessler RC The epidemiology of obsessive-compulsive disorder in the National Comorbidity Survey Replication. Mol Psychiatry (2010) 15:53–63. 10.1038/mp.2008.94 18725912 PMC2797569

[3] Hollander E Kwon JH Stein DJ Broatch J Rowland CT Himelein CA Obsessive-compulsive and spectrum disorders: overview and quality of life issues. J Clin Psychiatry (1996) 57(Suppl 8):3–6. 8698678

[4] Fontenelle IS Fontenelle LF Borges MC Prazeres AM Range BP Mendlowicz MV Quality of life and symptom dimensions of patients with obsessive-compulsive disorder. Psychiatry Res (2010) 179:198–203. 10.1016/j.psychres.2009.04.005 20483484

[5] Murray CJL Lopez AD The Global Burden of Disease: a comprehensive assessment of mortality and disability. In: Diseases, Injuries and Risk Factors in 1990 and projected 2020. Cambridge MA: Harvard University Press (1996). p. 1–98.

[6] McKay D Sookman D Neziroglu F Wilhelm S Stein DJ Kyrios M Efficacy of cognitive-behavioral therapy for obsessive-compulsive disorder. Psychiatry Res (2015) 225:236–46. 10.1016/j.psychres.2015.02.004 25613661

[7] Christensen H Hadzi-Pavlovic D Andrews G Mattick R Behavior therapy and tricyclic medication in the treatment of obsessive-compulsive disorder: a quantitative review. J Consult Clin Psychol (1987) 55:701–11. 10.1037//0022-006x.55.5.701 3331632

[8] Abramowitz JS Effectiveness of psychological and pharmacological treatments for obsessive-compulsive disorder: a quantitative review. J Consult Clin Psychol (1997) 1997 65:44–52. 10.1037//0022-006x.65.1.44 9103733

[9] Fineberg NA Brown A Reghunandanan S Pampaloni I Evidence-based pharmacotherapy of obsessive-compulsive disorder. Int J Neuropsychopharmacol (2012) 15:1173–91. 10.1017/S1461145711001829 22226028

[10] Fineberg NA Reghunandanan S Simpson HB Phillips KA Richter MA Matthews K Obsessive-compulsive disorder (OCD): Practical strategies for pharmacological and somatic treatment in adults. Psychiatry Res (2015) 227:114–25. 10.1016/j.psychres.2014.12.003 25681005

[11] Soomro GM Altman D Rajagopal S Oakley-Browne M Selective serotonin re-uptake inhibitors (SSRIs) versus placebo for obsessive compulsive disorder (OCD). Cochrane Database Syst Rev (2008) 1:CD001765. 10.1002/14651858.CD001765.pub3 PMC7025764 18253995

[12] Shannahoff-Khalsa DS Beckett LR Clinical case report: efficacy of yogic techniques in the treatment of obsessive compulsive disorders. Int J Neurosci (1996) 85:1–17. 8727678 10.3109/00207459608986347

[13] Shannahoff-Khalsa D Ray LE Levine S Gallen CC Schwartz BJ Sidorowich JJ Randomized controlled trial of yogic meditation techniques for patients with obsessive compulsive disorders. CNS Spectrums: The Intern J Neuropsychiatric Med (1999) 4:34–46. 10.1017/s1092852900006805 18311106

[14] Fossaluza V Diniz JB Pereira Bde B Miguel EC Pereira CA Sequential allocation to balance prognostic factors in a psychiatric clinical trial. Clinics (Sao Paulo) (2009) 64:511–8. 10.1590/S1807-59322009000600005 PMC2705482 19578654

[15] Shannahoff-Khalsa DS Yogic techniques are effective in the treatment of obsessive compulsive disorders. In: Hollander, E, and Stein, D, editors. Obsessive-compulsive disorders: Diagnosis, etiology, and treatment. New York, NY: Marcel Dekker Inc. (1997). p. 283–329.

[16] Shannahoff-Khalsa DS Kundalini Yoga meditation techniques in the treatment of obsessive compulsive and OC spectrum disorders. Brief Treat Crisis Intervention (2003) 3:369–82.

[17] Shannahoff-Khalsa DS Kundalini Yoga meditation techniques in the treatment of obsessive compulsive and OC spectrum disorders. In: Albert, PD, and Roberts, R, editors. Social Workers’ Desk Reference., New York, NY: Oxford University Press (2008). p. 606–12.

[18] Shannahoff-Khalsa DS Kundalini yoga meditation: techniques specific for psychiatric disorders, couples therapy, and personal growth. New York, London: W. W. Norton & Co. Inc. (2006).

[19] Shannahoff-Khalsa D Sacred Therapies: The kundalini yoga meditation handbook for mental health. New York, London: W. W. Norton, Co., Inc. (2012).

[20] Benson H Klipper MZ The relaxation response. New York: Harper Torch (2000).

[21] Goodman WK Price LH Rasmussen SA Mazure C Delgado P Heninger GR The Yale-brown obsessive compulsive scale. II. Validity Arch Gen Psychiatry (1989) 46:1012–6. 10.1001/archpsyc.1989.01810110054008 2510699

[22] Goodman WK Price LH Rasmussen SA Mazure C Fleischmann RL Hill CL The Yale-brown obsessive compulsive scale. I. Development, use, and reliability. Arch Gen Psychiatry (1989) 46:1006–11. 10.1001/archpsyc.1989.01810110048007 2684084

[23] Rosario-Campos MC Miguel EC Quatrano S Chacon P Ferrao Y Findley D The Dimensional yale-brown obsessive–compulsive scale (DY-BOCS): an instrument for assessing obsessive–compulsive symptom dimensions. Mol Psychiatry (2006) 11:495–504. 16432526 10.1038/sj.mp.4001798

[24] Guy W Clinical global impression. In: ECDEU Assessment manual for psychopharmacology. National Institute of Mental Health, P.H.S. US Department of Health and Human Services, Alcohol Drug Abuse and Mental Health Administration, NIMH Psychopharmacology Research Branch., Rockville, MD: P.H.S. US Department of Health and Human Services (1976).

[25] McNair D Lorr M Droppleman L Profile of moods scale (revised 1992). Educational and industrial testing service. San Diego, CA: Educational and Industrial Testing Services (1992).

[26] Beck AT Epstein N Brown G Steer RA An inventory for measuring clinical anxiety: psychometric properties. J Consult Clin Psychol (1988) 56:893–7. 10.1037//0022-006x.56.6.893 3204199

[27] Beck AT Ward CH Mendelson M Mock J Erbaugh J An inventory for measuring depression. Arch Gen Psychiatry (1961) 4:561–71. 10.1001/archpsyc.1961.01710120031004 13688369

[28] Cunha J Manual da versa o em Portugue’s das escalas de Beck. Sao Paulo: Casa do Psico’ logo (2001).

[29] Ware JE Kosinski M Dewey JE How to score version two of the SF-36 health survey. Lincoln, RI: QualityMetric, Incorporated (2000).

[30] Levine J Schooler NR SAFTEE: a technique for the systematic assessment of side effects in clinical trials. Psychopharmacol Bull (1986) 22:343–81. 3774930

[31] Bloch MH Bartley CA Zipperer L Jakubovski E Landeros-Weisenberger A Pittenger C Meta-analysis: hoarding symptoms associated with poor treatment outcome in obsessive-compulsive disorder. Mol Psychiatry (2014) 19:1025–30. 10.1038/mp.2014.50 PMC4169729 24912494

[32] Shannahoff-Khalsa DS Stress technology medicine, a new paradigm for stress and considerations for self-regulation. In: Brown, M, Koob, G, and Rivier, C, editors. Stress: Neurobiology and Neuroendocrinology. New York: Marcel Dekker Inc. (1991). p. 647–86.

[33] Shannahoff-Khalsa D Meditation: the science and the art. In: Ramachandran, VS, editor. The encyclopedia of human behavior. San Diego, CA: Academic Press (2012). p. 576–84.

[34] Shannahoff-Khalsa DS Selective unilateral autonomic activation: implications for psychiatry. CNS Spectrum: The Intern J Neuropsychiatric Med (2007) 12:625–34. 10.1017/s1092852900021428 17667891

[35] Shannahoff-Khalsa DS Psychophysiological states: the ultradian dynamics of mind-body interactions. in International Review of Neurobiology. London, New York, San Diego: Academic Press/Elsevier (2008). 10.1016/S0074-7742(07)80001-8 17967615

[36] Bhat S Varambally S Karmani S Govindaraj R Gangadhar BN Designing and validation of a yoga-based intervention for schizophrenia. Int Rev Psychiatry (2016) 28:327–33. 10.3109/09540261.2016.1170001 27117898

[37] Key BL Rowa K Bieling P McCabe R Pawluk EJ Mindfulness-based cognitive therapy as an augmentation treatment for obsessive-compulsive disorder. Clin Psychol Psychother (2017) 24:1109–20. 10.1002/cpp.2076 28194835

