# Supplementary material for: Kundalini Yoga Meditation Versus the Relaxation Response Meditation for Treating Adults With Obsessive-Compulsive Disorder: A Randomized Clinical Trial
Source: Front Psychiatry. 2019 Nov 11;10:793. doi: 10.3389/fpsyt.2019.00793 (PMC6859828; doi:10.3389/fpsyt.2019.00793)
Supplement: Supplementary file 1 [file Presentation_1.pdf]

Supplementary Material

Outline for Content:

1) Supplementary Methods: Calculation formula for the Bayesian Analysis
2) Results: Table S1 – CGI Scale Statistical Homogeny Exact Test
3) Results: Secondary Outcome Scales - Phase Two (4.5-months to 16.5-months) for DY-BOCS, POMS, BAI, BDI, and SF-36 (Figures S3-7).
4) Results: Bayesian Analysis figures for Posterior Density π of the population proportions of positive respondents, *P.* (See DY-BOCS Figure S8, POMS Figure S9, BAI Figure S10, BDI Figure S11, and SF-36 Figure S12)
5) The Kundalini Yoga Meditation Protocol and the Relaxation Response Meditation Protocol
5a.) The Kundalini Yoga Meditation Protocol Specific for OCD:
6.b.) The Relaxation Response Meditation Protocol:

Numbered Sections of Outline:

1) Supplementary Methods: Calculation formula for the Bayesian Analysis

The posterior density of P, the population proportion of positive respondents, and the probabilities of interest are as follows:

For X being the observed frequency of positive respondents the parameters of the posterior density functions, considering uniform priors, are $\alpha = X+1$ and $\beta = n-X+1$, n being the sample size. In this way, the posterior density can be written as:

$\mathbf{f}(\boldsymbol{P} \mid \mathbf{X} = \mathbf{x}) = (x!(n-x)!)/(n+1)!\ \boldsymbol{P}^{\alpha}\,(\mathbf{1} - \mathbf{P})^{\beta}$ for A! being the factorial of A. Hence, the probability of interest is $\boldsymbol{\pi}(\boldsymbol{p} \mid \mathbf{X} = \mathbf{x}) = \int_{p}^{1} f(P|X=x)dP = \mathbf{Pr}(\boldsymbol{P} \geq \boldsymbol{p} | \mathbf{X} = \mathbf{x})$.

2) Results: Table S1 – CGI Scale Statistical Homogeny Exact Test

CGI Frequencies in Phase 1

Arms	CGI Scores	
	1	2, 3 & 4
KY	8	7
RR	0	11

Exact p-value = 0.0067437

CGI Frequencies in Phase 1

Arms	CGI Scores	
	4	1, 2 & 3
KY	1	14
RR	4	7

Exact p-value = 0.0344345

CGI Frequencies in Phase 2

Arms	CGI Scores	
	1	2, 3 & 4
KY	3	5
RR	3	6

Exact p-value = 0.5838824

CGI Frequencies in Phase 2

Arms	CGI Scores	
	4	1, 2 & 3
KY	2	6
RR	3	6

Exact p-value = 0.4347878

Table S1 shows the Phase Two results for the CGI scale for the 17 patients that completed at least the first CGI scale measure, i.e., the 8.5-month mark (the 4-month mark of KY merged group therapy in Phase 2). Table S1 shows the frequencies in Phase Two for the possible scores of 1-4 for KY and RR. The number of 1's scored was 3 for both KY and RR, and the observation of a 2, 3, or 4 was 5 for KY and 6 for RR. The exact *p*-value was 0.584 indicating that there were no differences for the two subsets of patients when scoring for 1's. When comparing the two subgroups for the scores of 4, vs. a 1, 2, or 3, there were two 4's for KY and three for RR and 6 in each for a 1, 2, or 3. The exact *p*-value = 0.435, indicates again no subgroup differences. Histogram plots in Figure 5 show the frequency and relative % frequency for Phase Two with both groups separately. While the relative frequency appears to be slightly better for the initial KY patients, those differences are far from significant.

3) Results: Secondary Outcome Scales - Phase Two (4.5-months to 16.5-months) for DY-BOCS, POMS, BAI, BDI, and SF-36 (Figures S1-5).

Figures S1-5 are for the DY-BOCS, POMS, BAI, BDI, and SF-36, respectively. These 5 Supplementary Figures show the 8.5-month (*n*=17), 12.5-month (*n*=9), and 16.5-month (*n*=7) means when compared to their 4.5-month mean scores for those from their original Phase One groups separately and all patients combined in the Phase Two KY group.

Figure S1 shows the DY-BOCS mean % change improvement. For the 7 completing 16.5-months, there were 3 from the original KY group and 4 from the RR group. The mean % change improvement for the 7 subjects when comparing their 4.5-month and 16.5-month means was 26.22% (SD ±37.23%, *n*=7), with a 38.96% (SD ±52.69%) improvement for the 3 from KY and 16.67% (SD ±11.43%) for the 4 from Relaxation Response. The DY-BOCS mean % improvement for the 7 completers at 16.5-months compared to the Phase One 0-month baseline was 32.8% (SD ±36.11%). The 3 original KY improved by 51.02% (SD ±42.5%), and the 4 original RR patients by 20.79%, SD ±8.98%).

Figure S2 shows the POMS mean % change improvement for the 7 completers at 16.5-months compared to 4.5-months. The % improvement for the 7 completers was 21.13% (SD

+8.33%), with a 24.24% (SD +14.87%) improvement for the 3 from KY, and 18.81% (SD +23.44%) for the 4 from RR. The POMS % improvement for the 7 completers at 16.5-months compared to the Phase One baseline was 32.92% (SD +16.52%). The 3 original KY improved by 41.05% (SD +16.13%), and the 4 original RR patients by 26.83% (SD +14.0%).

Figure S3 shows the BAI mean % change improvement for the 7 completers at 16.5months compared to 4.5-months. The % improvement for the 7 completers was 8.03% (SD +53.46%), with a 24.12% (SD +54.43%) improvement for the 3 from KY, and -4.05% (SD +49.38%) for the 4 from RR. The BAI % improvement for the 7 completers at 16.5-months compared to the Phase One baseline was 2.11% (SD +86.42%). The 3 original KY improved by 34.29% (SD +38.14%), and the 4 original RR patients by -22.03% (SD +103.06%).

Figure S4 shows the BDI mean % change improvement for the 7 completers at 16.5-months compared to 4.5-months. The % improvement for the 7 completers was 45.28% (SD +36.49%). The 3 original KY patients improved by 36.19% (SD +42.68%), and the 4 original RR patients by 52.09% (SD +19.93%). The BDI % improvement for the 7 completers at 16.5-months compared to the Phase One baseline was 58.64% (SD +21.99%). The 3 original KY improved by 71.96% (SD +22.69%), and the 4 original RR by 48.65% (SD +15.08%).

Figure S5 shows the SF-36 mean % change improvement for the 7 completers at 16.5-months compared to 4.5-months. The % improvement for the 7 completers was 2.05% (SD +15.57%). The 3 original KY patients improved by 15.51% (SD +7.69%), and the original 4 RR patients by -8.05% (SD +11.93%). The BDI % improvement for the 7 completers at the 16.5-months compared to the Phase One baseline was 15.05% (SD +11.98%). The 3 original KY improved by 19.22% (SD +8.69%), and the 4 original RR by 11.92% (SD +13.1%).

The Phase Two results for the CGI scale are shown in Figure 5 and Table S1 for the 17 patients that completed at least the first CGI scale measure, i.e., the 8.5-month mark.

Figure S1. Phase One and Two DY-BOCS % Improvement

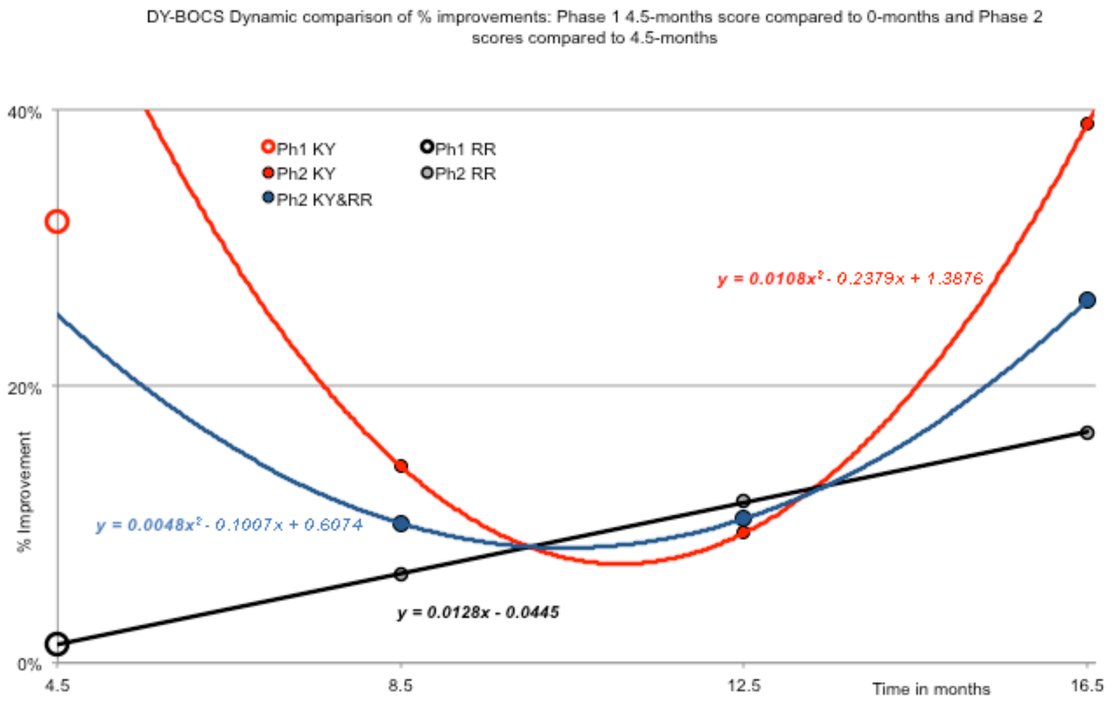

Figure S1 shows the mean % improvement plotted for the clinician administered Dimensional Yale-Brown Obsessive Compulsive Scale (DY-BOCS) Total Phase One Scores: 0-months vs. 4.5-months plotted on the y-axis for both the completers for the KY meditation group (n=15) and the RR meditation control group (n=11), with the KY mean in the open red circle, and the mean for RR at in the open black circle. The KY mean % change improvement was 31.969% (SD ±29.04%), RR 1.298% (SD ±18.6%). Group differences are significant ($f_{1.24}$ =9.384, p=0.005). Figure S1 also shows the Phase Two DY-BOCS mean % change improvement for the 7 when comparing their 4.5-month mean to their 16.5-month mean with a 26.22% (SD ±37.23%) improvement for the combined KY group therapy, and separately with a 38.96% (SD ±52.69%) improvement for the 3 originally from KY and 16.67% (SD ±11.43%) for the 4 originally from RR. The % improvement for the 7 completers at 16.5-months compared to their Phase One 0-month baselines was 33.56% (SD ±36.11%). For the 3 in the original KY group there was a 50.59% (SD ±49.27%) improvement, and 20.79% (SD ±8.98%) for the 4 RR patients.

Figure S2. Phase One and Two POMS TMD Scores: % Improvements

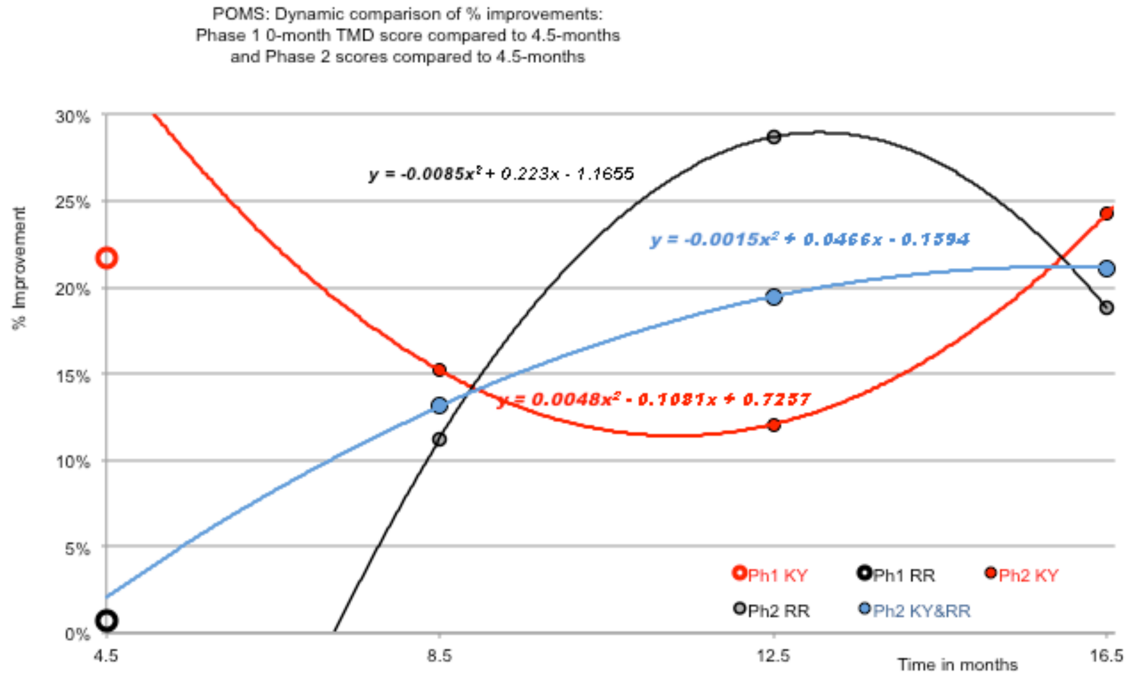

Figure S2 shows the mean % improvement plotted for the Profile of Moods Scale (POMS) scores for Phase One: 0-months vs. 4.5-months plotted on the y-axis for both the completers for the KY group (n=15) and the RR control group (n=11), with the KY mean in the open red circle, and the mean for RR at in the open black circle. The KY mean % change improvement was 39.123% (SD ±41.505%; n=15) and 3.869% (SD ±23.138%; N=11) for RR. Group differences are significant ($f_{1.25}$ = 6.42, p=0.018). Figure S2 also shows the Phase Two POMS % improvement for the 7 when comparing their 4.5-month mean to their 16.5-month mean. For all 7 patients combined the mean % change

improvement was 21.13% (SD ±20.40%), and a 24.24% (SD ±14.87%) improvement for the 3 originally from KY and 18.81% (SD ±23.44%) for the 4 originally from RR. The POMS % improvement for the 7 completers at 16.5-months compared to Phase One 0-month baseline was 32.92% (SD ±16.52%), and for the 3 originally in KY 41.05% (SD ±16.13%), and 26.83% (SD ±14.0%) for the 4 originally in RR.

Figure S3. Phase One and Two BAI Score % Improvement

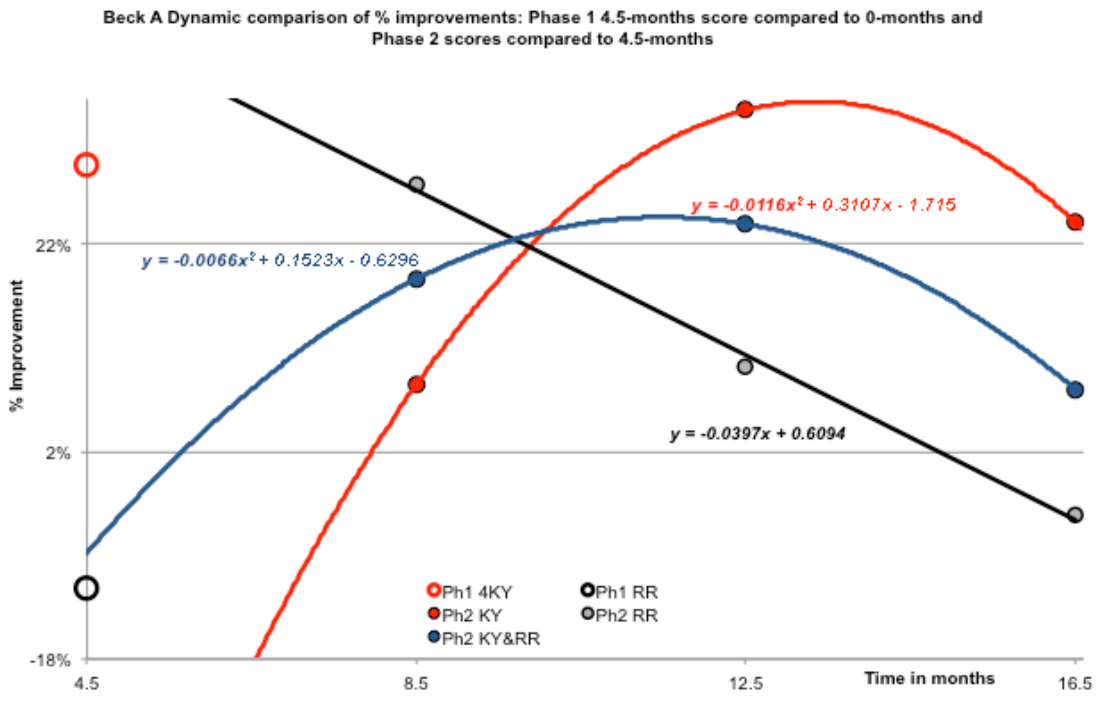

Figure S3 shows the mean % improvement plotted for the Beck Anxiety Inventory (BAI) scores for Phase One: 0-months vs. 4.5-months plotted on the y-axis for both the completers for the KY meditation group (n=15) and the RR meditation control group (n=11), with the KY mean in the open red circle, and the RR mean in the open black circle. The BAI KY mean % change improvement was 29.634% (SD ±36.799%; n=15) and -11.13% (SD ±64.429%; n=11) for RR. Group differences are significant ($f_{1.24} = 4.2$, p=0.05). Figure S3 also shows the Phase Two BAI mean % improvement for the 7 when comparing their 4.5-month mean to their 16.5-month mean. The mean for the all combined KY group was 8.03% (SD ±53.46%), with a 24.12% (SD ±54.43%) improvement for the 3 originally from KY and -4.05% (SD ±49.38%) for the 4 originally from RR. The BAI % improvement for the 7 completers at 16.5-months compared to the Phase One 0-month baseline was 2.11%, and for the 3 originally in KY the mean was 34.29% (SD ±38.14%), and -22.03% (SD ±103.06%) for the 4 originally in RR.

Figure S4. Phase One and Two BDI Score % Improvement

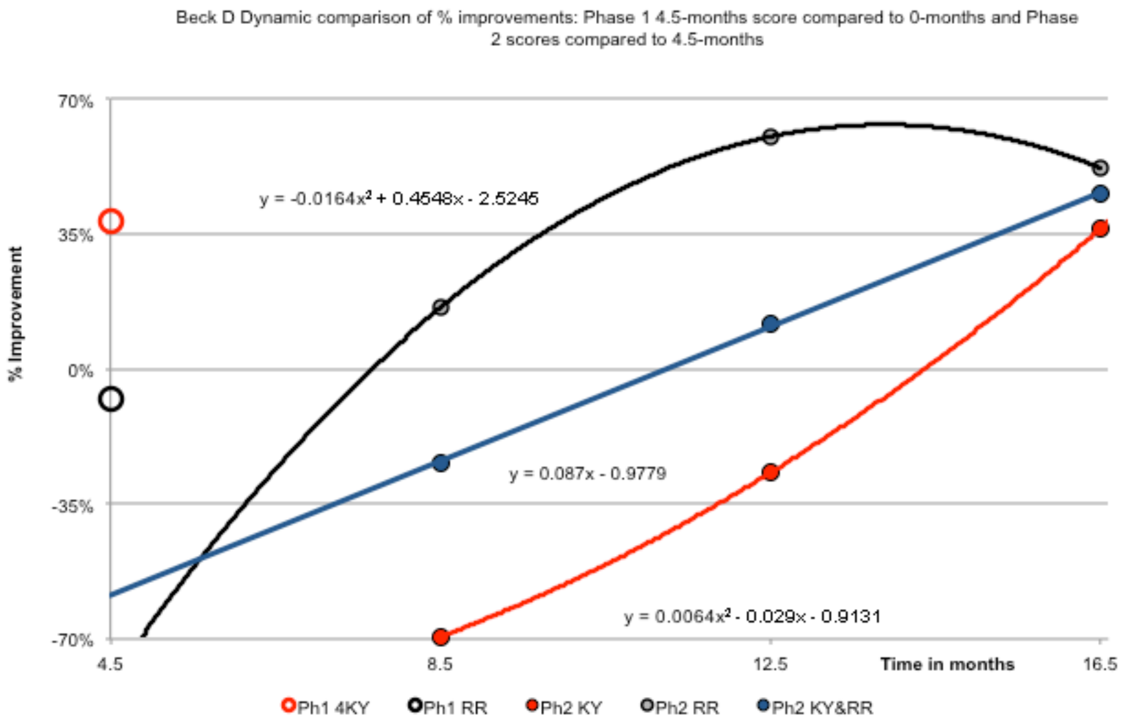

Figure S4 shows the mean % improvement plotted for the Beck Depression Inventory (BDI) scores for Phase One: 0-months vs. 4.5-months plotted on the y-axis for both the completers for the KY meditation group (n=15) and the RR meditation control group (n=11), with the KY mean in the open red circle, and the RR mean in the open black circle. The BDI KY mean % change improvement was 38.242% (SD $\pm$47.012%; n=15), and for RR -7.705% (SD $\pm$38.278%; n=11). Group differences are significant ($f_{1.24}$ = 7.05, p=0.014). Figure S4 also shows the Phase Two BDI % improvement for the 7 when comparing their 4.5-month mean to their 16.5-month mean. The combined KY therapy group mean was 45.28% (SD $\pm$36.49%), with a 36.19% (SD $\pm$42.68%) improvement for the 3 originally from KY and 52.09% (SD $\pm$19.93%) for the 4 originally from RR. The BDI % improvement (Figure S5) for the 7 completers at 16.5-months compared to their Phase One 0-month baseline was 58.64% (SD $\pm$21.99%), and for the 3 originally in KY the mean was 71.96% (SD $\pm$22.69), and 48.65% (SD $\pm$15.08%) for the 4 originally in RR.

Figure S5. Phase One and Two SF-36 score % Improvement

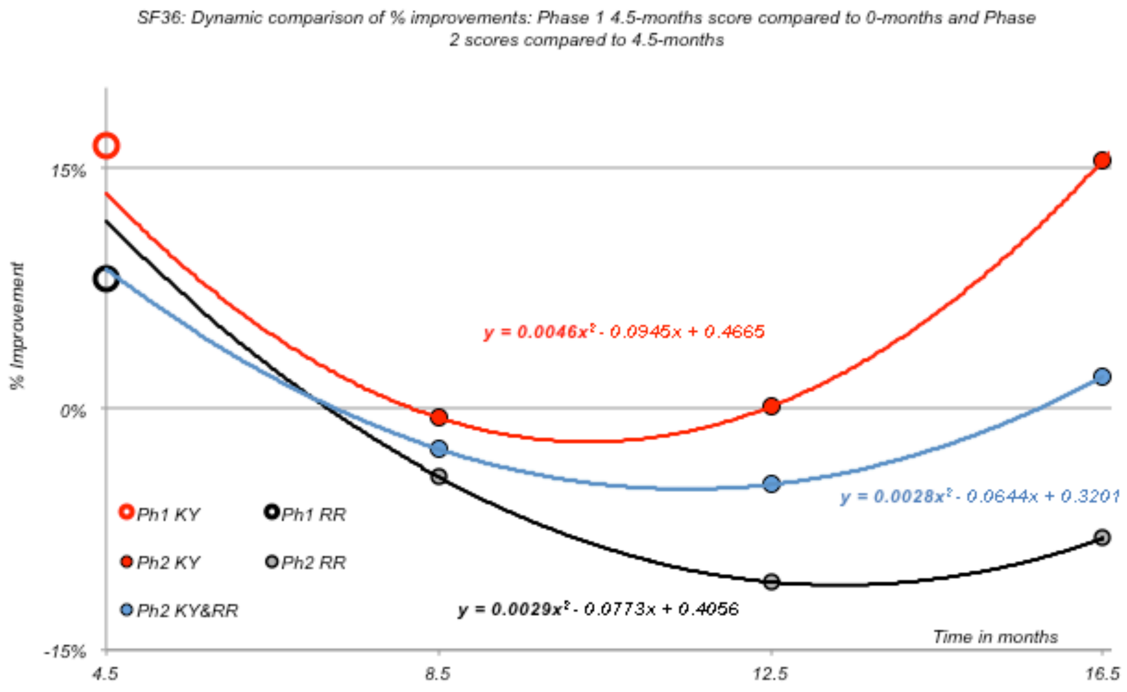

Figure S5 shows the mean % improvement plotted for the Short Form Health Survey (SF-36) scores for Phase One: 0-months vs. 4.5-months plotted on the y-axis for both the completers for the KY meditation group (n=15) and the RR meditation control group (n=11), with the KY mean in the open red circle, and the RR mean in the open black circle. The mean KY improvement was 16.4% (SD ±10.84%; n=15), RR 8.1% (SD ±5.98%; n=11). Group differences are not significant, p=0.18). Figure S5 also shows the Phase Two SF-36 % mean group improvement for the 7 when comparing their 4.5-month mean to their 16.5-month mean. The combined KY group mean was 2.05%, with a 15.51% (SD ±7.69%) improvement for the 3 originally from KY and -8.05% (SD +11.93%) for the 4 originally from RR. The SF-36 % improvement for the 7 completers at 16.5-months compared to their Phase One 0-month baseline was 15.05% (SD ±11.98%), and for the 3 originally in KY the mean was 19.22% (SD ±8.69%), and 11.92% (SD ±13.1%) for the 4 originally in RR.

4) Results: Bayesian Analysis figures for Posterior Density π of the population proportions of positive respondents, *P*. (See DY-BOCS Figure S6, POMS Figure S7, BAI Figure S8, BDI Figure S9, and SF-36 Figure S10)

A Bayesian statistical analysis for Phase Two was employed for the DY-BOCS, POMS, BAI, BDI, and SF-36 for the patients as a single group (KY + RR) comparing the 4.5-month value with their last measure taken at drop out, including the 7 that completed the trial at 16.5-months. The probability of greater than 50% of the patients improving to any extent, respectively, is 0.99364, 0.88106, 0.95187, 0.75966, 0.40726, for the five instruments, where 0 = no patients improving and 1 = all patients improving.

Figure S6. DY-BOCS: Posterior Density π of the population proportions of positive respondents, *P*

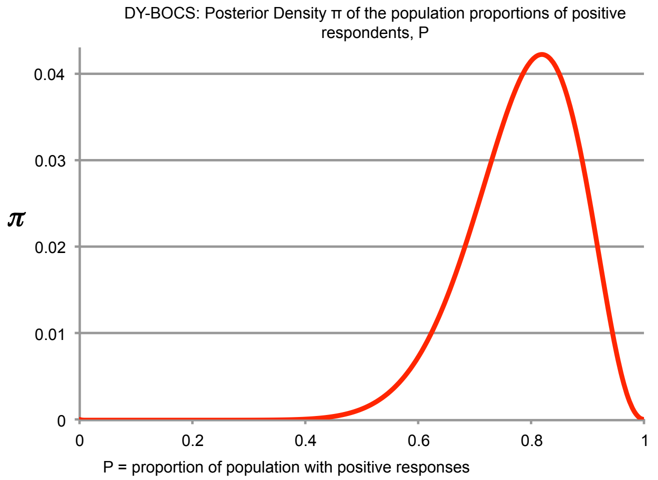

P	Probability
> 50%	0.99364
> 60%	0.95358
> 70%	0.79809
> 80%	0.45112
> 90%	0.08264

Phase 2: % of improvement	Ph 1 KY patients	100.0%	45.5%	-28.6%	100.0%	-20.0%	7.1%	12.5%	15.0%	
	Ph 1 RR patients	35.7%	11.1%	5.6%	14.3%	38.5%	5.0%	-26.3%	0.0%	0.0%

58

Figure S6. A Bayesian statistical analysis was performed for Phase Two for 17 patients comparing the 4.5-month value with their last DY-BOCS measure taken at dropout that included the 7 that completed the 16.5-months trial end point. This analysis showed that a 50% or greater probability criteria of patient improvement in the DY-BOCS in Phase Two was P=0.994, where 0 = no patients improving and 1 = all patients improving.

Figure S7. POMS: Posterior Density π of the population proportions of positive respondents, *P*

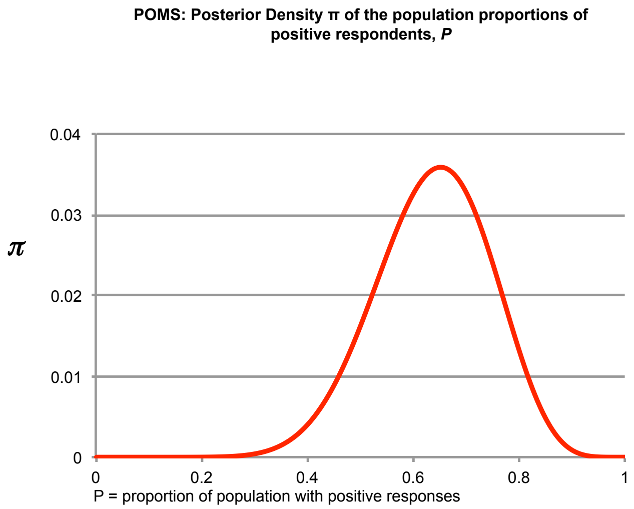

P	Probability
> 50%	0.88106
> 60%	0.62572
> 70%	0.27830
> 80%	0.05127
> 90%	0.00117

Phase 2: % of improvement	Ph 1 KY patients	41.5%	26.1%	5.2%	77.6%	-9.1%	-9.5%	30.4%	43.9%	
	Ph 1 RR patients	48.3%	17.5%	26.3%	-16.8%	14.9%	8.2%	-5.7%	-7.2%	-4.6%

59

Figure S7. A Bayesian statistical analysis was performed for Phase two for 17 patients comparing the 4.5-month value with their last POMS measure taken at dropout that included the 7 that completed the 16.5-months trial end point. This analysis showed that a 50% or greater probability criteria of patient improvement in the POMS in Phase Two was *P*=0.881, where 0 = no patients improving and 1 = all patients improving.

Figure S8. BAI: Posterior Density π of the population proportions of positive respondents, *P*

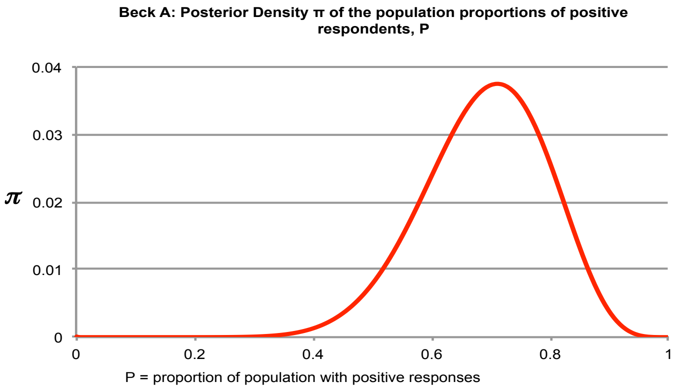

P	Probability
> 50%	0.95187
> 60%	0.79124
> 70%	0.46562
> 80%	0.13292
> 90%	0.00642

Phase 2: % of improvement	Ph 1 KY patients	85.7%	33.3%	-46.7%	33.3%	22.2%	-20.0%	-55.6%	34.8%	
	Ph 1 RR patients	75.0%	0.0%	-50.0%	-41.2%	66.7%	57.1%	0.0%	8.0%	6.3%

60

Figure F8. A Bayesian statistical analysis was performed for Phase Two for 17 patients comparing the 4.5-month value with their last BAI measure taken at dropout that included the 7 that completed the 16.5-months trial end point. This analysis showed that a 50% or greater probability criteria of patient improvement in the BAI in Phase Two was *P*=0.952, where 0 = no patients improving and 1 = all patients improving.

Figure S9. BDI: Posterior Density π of the population proportions of positive respondents, *P*

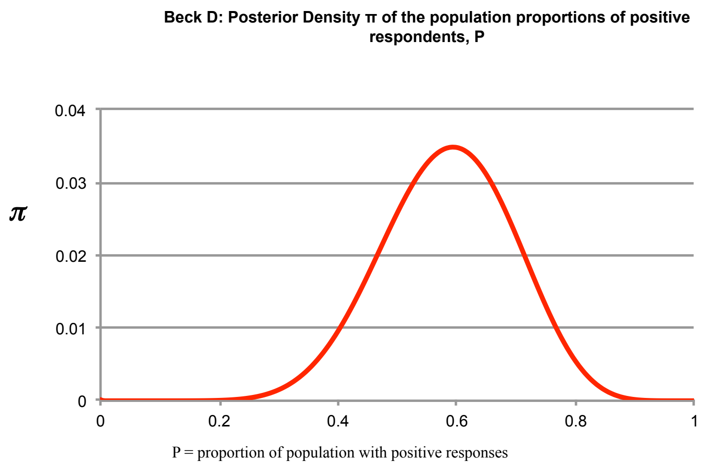

P	Probability
> 50%	0.75966
> 60%	0.43656
> 70%	0.14068
> 80%	0.01628
> 90%	0.00017

Phase 2: % of improvement	Ph 1 KY patients	100.0%	28.6%	-20.0%	80.0%	-166.7%	-200.0%	-3.6%	-50.0%	
	Ph 1 RR patients	80.0%	50.0%	54.5%	23.8%	0.0%	29.4%	-7.7%	-28.6%	18.2%

61

Figure S9. A Bayesian statistical analysis was performed for Phase Two for 17 patients comparing the 4.5-month value with their last BDI measure taken at dropout that included the 7 that completed the 16.5-months trial end point. This analysis showed that a 50% or greater probability criteria of patient improvement in the BDI in Phase Two was *P*=0.76, where 0 = no patients improving and 1 = all patients improving.

Figure S10. SF-36: Posterior Density π of the population proportions of positive respondents, *P*

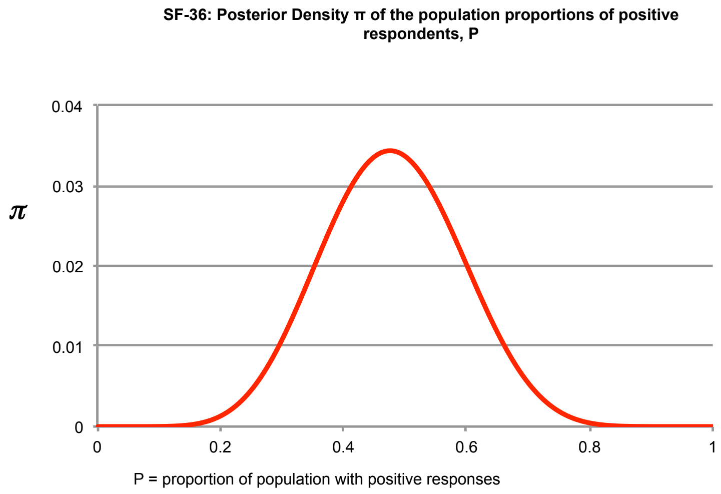

P	Probability
> 50%	0.40726
> 60%	0.13471
> 70%	0.02097
> 80%	0.00091
> 90%	0.00000

Phase 2: % of improvement	Ph 1 KY patients	26.3%	9.1%	11.1%	27.0%	-42.0%	-19.0%	-18.4%	26.5%	
	Ph 1 RR patients	-3.6%	9.0%	-22.6%	-15.0%	9.5%	-20.5%	7.8%	-19.3%	-16.5%

62

Figure F10. A Bayesian statistical analysis was performed for Phase 2 for 17 patients comparing the 4.5-month value with their last SF-36 measure taken at dropout that included the 7 that completed the 16.5-months trial end point. This analysis showed that a 50% or greater probability criteria of patient improvement in the SF-36 in Phase Two was *P*=0.407, where 0 = no patients improving and 1 = all patients improving.

5) The Kundalini Yoga Meditation Protocol and the Relaxation Response Meditation Protocol

5a.) The Kundalini Yoga Meditation Protocol Specific for OCD (Copyright © David Shannahoff-Khalsa, 1992. No portion of this protocol may be reproduced without the express written permission of the author.)

The entire KY protocol used in the uncontrolled study (Shannahoff-Khalsa & Beckett, 1996), the controlled study (Shannahoff-Khalsa, 1997; Shannahoff-Khalsa et al., 1999), and this RCT is described here in complete detail. All techniques in this protocol can be performed while sitting in a chair. All of the techniques taught here are from the KY tradition as taught by Yogi Bhajan.

This protocol includes eight primary techniques (1–8) to be used on a daily basis, and three additional techniques (9–11) to be used at personal discretion. This protocol was initially reported in complete detail in Shannahoff-Khalsa (1997).

1. To Induce a Meditative State, "Tuning In"

Sit with a straight spine and with the feet flat on the floor, if sitting in a chair. Put the hands together at the chest in "prayer pose"—the palms are pressed together with 10 to 15 lbs of pressure between the hands. The area where the sides of the thumbs touch rests on the sternum with the thumbs pointing up (along the sternum); the fingers are together and point up and out at a 60-degree angle to the ground. The eyes are closed and are focused at the "third eye" (imagine a sun rising on the horizon). A mantra is chanted out loud in a one-and-a-half breath cycle. Inhale first through the nose and chant "Ong Namo," with an equal emphasis on the Ong and the Namo. Then immediately follow with a half breath inhalation through the mouth and chant "Guru Dev Namo," with approximately equal emphasis on each word. The practitioner should experience the vibrations that these sounds create on the upper palate and throughout the cranium while letting the mind be carried by the sounds. This exercise should be repeated a minimum of 3 times; it was employed in therapy for about 10 to 12 times. This technique helps create a "meditative state of mind" and is highly recommended as a precursor to the other techniques.

2. Spine Flexing for Vitality

This technique can be practiced either while sitting in a chair or on the floor, in a cross legged position. If you are in a chair, hold the knees with both hands for support and leverage. If you are sitting cross-legged, grasp the ankles in front with both hands. Begin by pulling the chest up and forward, inhaling deep at the same time; then exhale as you relax the spine down into a slouching position. Keep the head up straight without allowing it to move much with the flexing action of the spine. This position helps prevent a whip action of the cervical vertebrae. All breathing should only be through the nose—both the inhale and exhale. The eyes are closed, as if you were looking at a central point on the horizon, the "third eye," otherwise described as the notch region on the nose exactly midway between the eyes. The mental focus is kept on the sound of the breath while listening to the fluid movement of the inhalation and exhalation. Begin the technique slowly while loosening up the spine. Eventually, a very rapid movement can be achieved with practice, reaching a rate of 1 to 2 times per second for the entire movement. A few minutes are sufficient in the beginning. Later, there is no time limit. Food should be avoided just before this exercise. If an unpleasant feeling of light-headedness develops, stop momentarily and then continue. Be careful; flex the spine slowly in the beginning. Relax for 1 to 2 min when finished.

3. Shoulder Shrugs for Vitality

While keeping the spine straight, rest the hands on the knees if sitting in a cross-legged position or with hands on the thighs if on a chair. Inhale and raise the shoulders up toward the ears; then exhale, letting them down. All breathing is done through the nose. Eyes should be kept closed and focused at the third eye. Mentally listen to the sound of the inhalation and exhalation. Continue this action rapidly, building to three times per second for a maximum of 2 min. This technique should not be practiced by individuals who are hyperactive.

4. Meditation Technique for Insanity—Technique for Reducing Anxiety, Stress, and Mental Tension

Sit and maintain a straight spine. Relax the arms and the hands in the lap. Focus the eyes on the tip of

the nose. You cannot see the end, just the sides of the nose, as they appear blurred while focusing on the tip. Open the mouth as wide as possible, slightly stressing the temporal mandibular joint; touch the tongue tip to the upper palate where it is hard and smooth in the center. Breathe continuously through the nose only, making the respiration slow and deep. Let the mental focus be on the sound of the breath; listen to the sound of the inhale and exhale. Maintain this pattern for at least 3 to 5 min with a maximum of 8 min on the first trial. With practice it can be built up to 31 min, maximum. This technique was originally taught as a meditation for insanity; it curbs a restless mind, it brings stillness and mental quiet.

5. Technique for Reducing Anxiety, Stress, and Mental Tension

Sit and maintain a straight spine. The hands are in front of the chest at heart level. The left hand is 2 inches from the chest, and the right is about 2 inches behind the left (4 inches from the chest); the left fingers point to the right. The right palm faces the back of the left hand with fingers pointing to the left. The thumbs of both hands point up straight but are not pulled back tightly. The thumbs are in a relaxed upward posture. The eyes are open and focused on the tip of the nose. The breathing pattern is through the nose only. Inhale, then keep the breath in as long as possible; then exhale and keep the breath out as long as possible, without creating undo discomfort at any stage. When finished, inhale maintaining the eye and-hand posture; then tense every muscle in the body for about 10 sec, exhale and repeat two times. Build the capacity for this technique to a maximum time of 15 min. Avoid this exercise if you have high blood pressure or are pregnant. This technique was taught for relaxing the mind in response to emotional stress and mental tension, and the following technique was taught to be complementary to its practice.

6. Technique for Reducing Anxiety, Stress, and Mental Tension

Sit as noted in the previous technique. Eyes are open and focused on the tip of the nose during the entire exercise. Attempt to pull the nose down toward the upper lip by actually pulling the upper lip down over the upper front teeth using the muscles of the upper lip. The mouth is left open during this exercise with the constant tension on the upper lip. This exercise has three steps. 1. Start with the hands and arms up at 45 to 60 degrees; inhale deeply; tightly clench the fists and pull them down toward the abdomen. 2. Keep the breath in, the eyes focused, and the lip pulled; maintain tension in the fists; bring the shoulders up toward the ears, tensing them as they go up. 3. Exhale and relax, but keep the lip pulled down and the eyes at the tip of nose. Repeat the entire exercise six times. Avoid this exercise if you have high blood pressure or are pregnant. This short exercise is claimed to be so effective that, if done correctly, it can relieve the most tense person.

7. Technique for Managing Fears

Sit with a straight spine. Close the eyes. Place the left hand into the navel point, with the four finger tips and thumb grouped together, and press very lightly. Place the four fingers of the right hand (pointing left) over the third eye (on the forehead just above the root of the nose), as if feeling your temperature. Play the tape of Chattra Chakra Vartee by Wahe Guru Kaur (1986) or by Kulwant Singh in Healing Sounds of the Ancients Collection Volume One (2002) for 3 min while assessing your fears and consciously relating to the mental experience of your fears. This technique is claimed to help manage acute states of fear and help eliminate fearful images and negative emotions that have developed due to fearful experiences. The effect is that the negative emotions related to specific fears are replaced with positive emotions, thereby slowly creating a new and different mental association

with the stimulus. This technique is analogous to the practice of exposure and response prevention. However, it is not necessary to actually physically engage the threat or feared substance.

8. Technique for OCD—The Obsessive-Compulsive Disorder Breath (OCDB)

Sit with a straight spine in a comfortable position, either with the legs crossed while sitting on the floor or in a straight back chair with both feet flat on the floor. Close the eyes. Use the right thumb tip to block the end of the right nostril; the other fingers point up straight; allow the arm to relax (elbow should not be creating unnecessary tension by sticking up and out to the side). A secure plug can also be used for the right nostril. Inhale slow and deep through the left nostril; hold in long; exhale slowly and completely through the same nostril (left nostril); hold out long. The mental focus should be on the sound of the breath. Continue this pattern with a maximum time of 31 min for each sitting. Initially, begin with a comfortable rate and time, but graduate to one where the effort presents a fair challenge for each phase of the breath. Deciding how long to hold the breath in or out varies from person to person. Ideal time per complete breath cycle is 1 minute, where each section of the cycle lasts exactly 15 sec. With daily discipline, this rate of respiration can be achieved within 5 to 6 months for the full 31 min. Yogic experiments (personal communication, Yogi Bhajan) claim that 90 days of 31 min per day, using the perfected rate of one breath per minute with 15 sec per phase, will completely eliminate all OC disorders.

9. Meeting Mental Challenges—The "Victory Breath"

This technique can be used at any time. It does not require that the practitioner sit. It can be employed while driving a car, while in a conversation, while taking a test, and so forth. The eyes can be open or closed, depending on the situation. Take a deep breath through the nose, and hold this breath over 3 to 4 sec. During the hold phase, mentally hear the three syllables (sounds) of the word "victory" (vic-tor-ee), then exhale. Mentally creating the three sounds should take 3 to 4 sec, not longer and not less. The entire time of each repetition should be about 10 sec. This technique can be employed multiple times, until the patient achieves the desired relief. When employed in the therapy sessions, the technique was usually done for 3 to 5 min, with the eyes closed while sitting with a straight spine to maximize the effects. This technique is very helpful as a "thought stopping" technique, for a patient "on the go." There is no time limit to its practice. It can be used to help reduce obsessive thoughts and resist the urge to perform compulsive rituals. Most patients found this technique very useful and a great tool for an active day. This technique can be used at any time a person feels mentally challenged.

10. Chant to Turn Negative Thoughts into Positive Thoughts

This technique should be employed in a peaceful environment while sitting with a straight spine and with the eyes closed. The mantra "Ek Ong Kar Sat Gurprasad Sat Gurprasad Ek Ong Kar" is repeated a minimum of five times. It can be practiced from 5 to 11 min, while chanting it rapidly with up to five repetitions per breath. Eventually, one no longer thinks about the order of the sounds; they come automatically. The mental focus should be on the vibration created against the upper palate and throughout the cranium. If performed correctly, a very peaceful, bright, elevated, and "healed" state of mind is achieved, especially when the practitioner reaches the 11-min time with five repetitions per breath.

11. Technique for Anger

Sit with a straight spine, and close the eyes. Simply chant out loud “Jeeo, Jeeo, Jeeo, Jeeo” continuously and rapidly for 11 min without stopping (pronounced like the names for the letters G and O). During continuous chanting, you do not stop to take long breaths, but you do continue with just enough short breaths to keep the sound going. Eleven minutes is both the minimum and maximum time for this technique. This technique is useful even for a “red hot” angry mind, and the effects can last up to 3 days, depending on the severity of the anger. Practicing twice a day or more is acceptable for the most severe states.

5.b.) The Relaxation Response Meditation Protocol:

The revised version of the RR[20] was taught here (see Benson, H., Klipper, M.Z., 2000. The Relaxation Response. Harper Torch, New York). This version and the original RR (Benson H. The Relaxation Response. New York: Morrow; 1975.) were devised by Herbert Benson at Harvard. The revised instructions included the following: Sit in a comfortable and stable position with a straight spine, distributing the body weight equally in the hip area, place the legs with an approximately 90º angle between the thighs and calves, with the feet flat on the ground, with the palms resting comfortably in the lap. Place the shoulders in a "neutral" way, avoiding excessive forward or backward rotation, and take out the spinal stability. The head is held straight distributing the weight equally over the cervical spine. The eyes are closed, and the patient begins a process of body positioning awareness, starting with the feet, then the legs, thighs, hips, abdomen, spine, shoulders, arms, forearms, hands, and head. This is followed by fifteen cycles of abdominal breathing. Then the patient is instructed to consciously observe the breath entering through the nose following the path to the lungs and then expiring through the nose. At the end of each exhalation, the word "One" (translated to Portuguese: “UM”) is repeated. They are instructed to ignore with a nonjudgmental attitude, the scattered and intrusive thoughts that distracts their attention from the mental repetition of the word "One" and the flow of air, and to bring their attention back to the sound and the breath when they noticed their loss of attention. This was practiced for three rounds of 20 min in the group. They were instructed to practice the RR for 60 min at home every day.
